# Efficacy and Safety of Linaclotide as an Adjunct to Polyethylene Glycol in Bowel Preparation: A Meta‐Analysis

**DOI:** 10.1111/1751-2980.70008

**Published:** 2025-09-16

**Authors:** Muhammad Shahzil, Fariha Hasan, Ali Akram Qureshi, Zainab Jamil, Talha Kashif, Muhammad Saad Faisal, Taha Bin Arif, Ammad Javaid Chaudhary, Umer Farooq, Hassam Ali, John M. Levenick

**Affiliations:** ^1^ Department of Internal Medicine Penn State Health Milton S. Hershey Medical Center, The Pennsylvania State University Hershey Pennsylvania USA; ^2^ Department of Internal Medicine Cooper University Hospital Camden New Jersey USA; ^3^ Department of Medicine King Edward Medical University Lahore Punjab Pakistan; ^4^ Department of Internal Medicine Henry Ford Hospital Detroit Michigan USA; ^5^ Department of Internal Medicine Sinai Hospital of Baltimore Baltimore Maryland USA; ^6^ Division of Gastroenterology & Hepatology Saint Louis University School of Medicine St. Louis Missouri USA; ^7^ Department of Gastroenterology ECU Health Greenville North Carolina USA; ^8^ Department of Gastroenterology and Hepatology Penn State Health Milton S. Hershey Medical Center, The Pennsylvania State University Hershey Pennsylvania USA

**Keywords:** bowel preparation, colonoscopy, colorectal neoplasms, linaclotide, polyethylene glycols

## Abstract

**Objectives:**

Linaclotide, a guanylyl cyclase‐C agonist, may enhance efficacy and tolerability when combined with polyethylene glycol (PEG) for bowel preparation. This meta‐analysis evaluated linaclotide plus PEG versus PEG alone for bowel preparation prior to colonoscopy.

**Methods:**

Randomized controlled trials (RCTs) including adults undergoing colonoscopy that compared linaclotide plus PEG with PEG alone for bowel preparation were identified via database search up to March 2024. Statistical analysis was performed in RevMan Web using random‐effects models.

**Results:**

Eleven RCTs were analyzed. Adequate bowel preparation rate was comparable (risk ratio [RR] 1.01, 95% confidence interval [CI] 0.98–1.04; *I* ^2^ = 23%), as was cecal intubation rate (RR 1.01, 95% CI 1.00–1.01). Subgroup analyses showed that compared with 3‐L PEG alone, 2‐L PEG plus linaclotide was non‐inferior, while 3‐L PEG plus linaclotide was superior regarding bowel preparation adequacy (RR 1.11, 95% CI 1.01–1.23) and total Boston Bowel Preparation Scale (BBPS) score (mean difference 0.44, 95% CI 0.04–0.85). Right and left colon BBPS scores were also higher with linaclotide. Polyp detection rate improved significantly in the 3‐L PEG plus linaclotide subgroup (RR 1.78, 95% CI 1.32–2.40), whereas adenoma detection rate and withdrawal time were comparable. Linaclotide reduced abdominal pain, bloating, nausea, and sleep disturbance, and increased willingness to repeat colonoscopy.

**Conclusions:**

Linaclotide with PEG provides comparable overall bowel cleansing to PEG alone while reducing adverse events and improving patient acceptance. Importantly, 2‐L PEG plus linaclotide was non‐inferior compared with 3‐L PEG, whereas 3‐L PEG plus linaclotide showed superiority over 3‐L PEG alone, supporting its use in low‐volume bowel preparation strategies.

## Graphical abstract

Linaclotide combined with low‐volume polyethylene glycol (PEG) offers comparable bowel‐cleansing efficacy to traditional high‐volume PEG regimen while improving patient tolerability. Key benefits include higher segmental Boston Bowel Preparation Scale (BBPS) scores, reduced adverse events, and increased willingness to repeat the colonoscopic procedure, highlighting its potential as a patient‐friendly alternative for colonoscopy preparation. PRISMA, Preferred Reporting Items for Systematic reviews and Meta‐Analyses; RR, risk ratio.

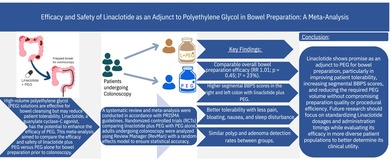

## 1 Introduction

Colonoscopy, acting as a gastrointestinal (GI) interventional procedure, is hailed for its diagnostic and therapeutic efficacy. The most important indication for colonoscopy is the diagnosis of colorectal cancer (CRC) and its precancerous lesions [1]. CRC is the third most common cause of cancer‐related mortality in the United States [2]. A previous meta‐analysis including 1.4 million patients has suggested that colonoscopy screening is associated with 89% and 64% decrease in CRC incidence and mortality, respectively [3]. This protective effect is linked to the success of colonoscopy, which depends directly on the adequacy of bowel preparation. Inadequate bowel preparation impacts the diagnostic accuracy of colonoscopy, which significantly reduces the lesion detection rate and increases the chance of cecal intubation failure [4, 5]. In addition to reducing the diagnostic and therapeutic potential, other metrics such as patient‐reported pain are also worse when bowel preparation is inadequate [6]. Therefore, the European Society of Gastrointestinal Endoscopy (ESGE) recommends a minimum of 90% adequate bowel preparation before colonoscopic examination [4]. Various oral laxatives have been explored in search of optimal results, especially when considering the cleansing efficacy. Polyethylene glycol (PEG), magnesium citrate, magnesium hydroxide, bisacodyl, and sodium picosulfate are some of the explored options [1]. Of them, 4‐L PEG is the most commonly used regimen; however, the use of large amounts of laxative solution has an overall low tolerability [1, 7]. Efforts have been made to use a low‐volume bowel preparation with 2‐L PEG along with another adjuvant substance to achieve the same results as high‐volume PEG [7]. One such adjuvant approach is the application of linaclotide combined with 2‐L PEG for bowel cleansing. Linaclotide has been approved by the U.S. Food and Drug Administration (FDA) for constipation associated with constipation‐predominant irritable bowel syndrome (IBS) or chronic idiopathic constipation [8]. It is a novel agonist of guanylyl cyclase‐2C (GC‐2C), which increases cyclic guanosine monophosphate (cGMP) levels in the colonic mucosa, thereby stimulating the secretion of chloride and bicarbonate into the intestinal lumen via activation of the cystic fibrosis transmembrane conductance regulator (CFTR) ion channel and resulting in increased intestinal fluid secretion and accelerated GI transit. Along with its laxative effects, linaclotide also improves abdominal pain via increased extracellular cGMP level in the submucosa, which inhibits the colonic nociceptors [8]. The effects of linaclotide have recently received significant attention as a possible way to mitigate the side effects of large‐volume PEG. Furthermore, a nonrandomized, historically controlled trial has shown encouraging results, where single‐dose linaclotide with PEG was found to be equal to 4‐L PEG in terms of bowel cleansing efficacy and visualization scores, as well as the small bowel transit time (SBTT) [9]. We conducted this meta‐analysis aiming to compile the available data and explore the pooled estimates of important outcomes related to the use of linaclotide combined with PEG as compared with the traditional use of PEG alone for bowel preparation before colonoscopy.

## 2 Materials and Methods

This meta‐analysis was conducted in accordance with the *Cochrane Handbook for Systematic Reviews of Interventions* and adhered to the Preferred Reporting Items for Systematic reviews and Meta‐Analyses (PRISMA) statement [10, 11]. This study was registered with the International Platform of Registered Systematic Review and Meta‐analysis Protocols (INPLASY) (no. INPLASY2024120123). Ethical approval was not required, as the analysis was performed based on previously published data.

### 2.1 Search Strategy

A comprehensive literature search was conducted across multiple databases, including the Cochrane Central Register of Controlled Trials (CENTRAL), Web of Science, MEDLINE (via PubMed), and EMBASE (Elsevier), covering all articles published from database inception to March 2024 without language restriction. The search specifically targeted randomized controlled trials (RCTs) evaluating linaclotide in combination with PEG for bowel preparation prior to colonoscopy. Both Medical Subject Headings (MeSH) and free‐text terms were used for the literature search, including “Linaclotide,” “Linzess,” “Polyethylene Glycol,” “Bowel Preparation,” and “Colonoscopy.” In addition, ClinicalTrials.org, World Health Organization (WHO) International Clinical Trials Registry Platform (ICTRP), and the ProQuest Dissertations were also searched, and the reference lists of the identified articles were reviewed for additional pertinent studies. A detailed search strategy, including specific search strings, is provided in Supporting Information.

### 2.2 Inclusion and Exclusion Criteria

Studies considered for the meta‐analysis were RCTs on the comparison of linaclotide combined with PEG with standard PEG‐based regimens for bowel preparation. The inclusion criteria were as follows: (i) studies including adult participants aged 18 years or older who underwent colonoscopy; (ii) studies comparing the efficacy and safety between the intervention group involving linaclotide combined with PEG (1, 2, or 3 L) and a control group receiving PEG (1, 2, or 3 L) alone for bowel preparation; and (iii) those reporting at least one relevant outcome, including adequate bowel preparation, Boston Bowel Preparation Scale (BBPS) scores, cecal intubation time, and cecal intubation rate. Studies focusing on patients with chronic constipation were excluded. Case reports, case series with fewer than 10 patients, single‐arm studies, clinical guidelines or consensus, non‐comparative studies, reviews, meta‐analyses, animal studies, conference abstracts, observational studies, and unpublished data were excluded. In duplicates identified from overlapping datasets, preference was given to the most recent and comprehensive publication.

### 2.3 Selection Process

Records identified from the databases were imported into Mendeley version 1.19.8 (Elsevier, Amsterdam, The Netherlands) for de‐duplication. Two reviewers (M.S. and A.A.Q.) independently screened titles and abstracts for relevance, followed by full‐text assessments based on the eligibility criteria. Discrepancies were resolved through discussion or consultation with a third reviewer (A.J.C.).

### 2.4 Data Extraction

Data extraction was performed by two authors (M.S. and F.H.) independently in a standardized manner using a predefined Excel template (Microsoft, Seattle, WA, USA). Discrepancies were resolved by consensus or discussion with a third reviewer (A.J.C.). The following data were extracted from each study: name of the first author, year of publication, study design, country of the population studied, study design, study size and number of cases, types and dosages of bowel preparation regimens used in the interventional and control groups, characteristics of the participants (age, gender, body mass index [BMI], etc.), outcome data, and study characteristics based on the PICOS (Population, Intervention, Comparison, Outcomes, and Study) framework. Articles published in languages other than English were translated using Google Translate, ChatGPT, and DeepL Translator to ensure accuracy and minimize discrepancies.

### 2.5 Outcomes

The primary outcomes included adequate bowel preparation, BBPS scores, cecal intubation rate, and cecal intubation time. The secondary outcomes included withdrawal time, adverse events, adenoma detection rate (ADR), polyp detection rate (PDR), and participants' willingness to repeat the procedure.

### 2.6 Risk of Bias Assessment

The risk of bias of the included RCTs was evaluated using the revised Cochrane Risk of Bias Tool 2.0 for RCTs (RoB 2.0). This tool examines bias arising from the randomization process, deviations from intended interventions, missing outcome data, measurement of the outcome, and selection of the reported result.

The certainty of evidence for each outcome was assessed using the GRADE framework [12], which evaluates study limitations, consistency of effect, imprecision, indirectness, and publication bias.

### 2.7 Statistical Analysis

Meta‐analyses were performed using RevMan Web (The Cochrane Collaboration, Copenhagen, Denmark) with a random‐effects model to account for variability among studies. Dichotomous outcomes were expressed as the risk ratio (RR) with the corresponding 95% confidence interval (CI), while continuous outcomes were presented as mean difference (MD) with 95% CI. A *p* value of less than 0.05 was considered statistically significant.

Heterogeneity among studies was assessed using the Chi‐square test (significance set at *p* < 0.10) and quantified with the Higgins *I* ^2^ statistic. *I* ^2^ values of 25%, 50%, and 75% were interpreted as representing low, moderate, and high heterogeneity, respectively. In cases of substantial heterogeneity (*I* ^2^ > 50%), potential sources were explored through subgroup and sensitivity analyses. All statistical analyses adhered to the guidelines provided in the *Cochrane Handbook for Systematic Reviews of Interventions* [11] and the methodological recommendations by Rücker et al. and Luo et al. [13, 14]. Continuous and dichotomous data were combined following the appropriate formulas and guidance to ensure accurate synthesis of the evidence.

Publication bias was assessed using funnel plots for outcomes reported in more than 10 studies. Sensitivity analyses were performed by excluding studies with a high risk of bias to evaluate the robustness of findings.

Initially, analyses were performed by comparing the linaclotide plus PEG group with the PEG‐alone group, regardless of their dosage. Subgroup analyses were subsequently conducted based on the volume of PEG used in the included studies. Subgroup analyses could not be performed based on linaclotide dosages because of the limited uniformity of data reported by studies using the same PEG volume in both the control and the intervention groups.

## 3 Results

### 3.1 Description of the Studies

**FIGURE 1:**
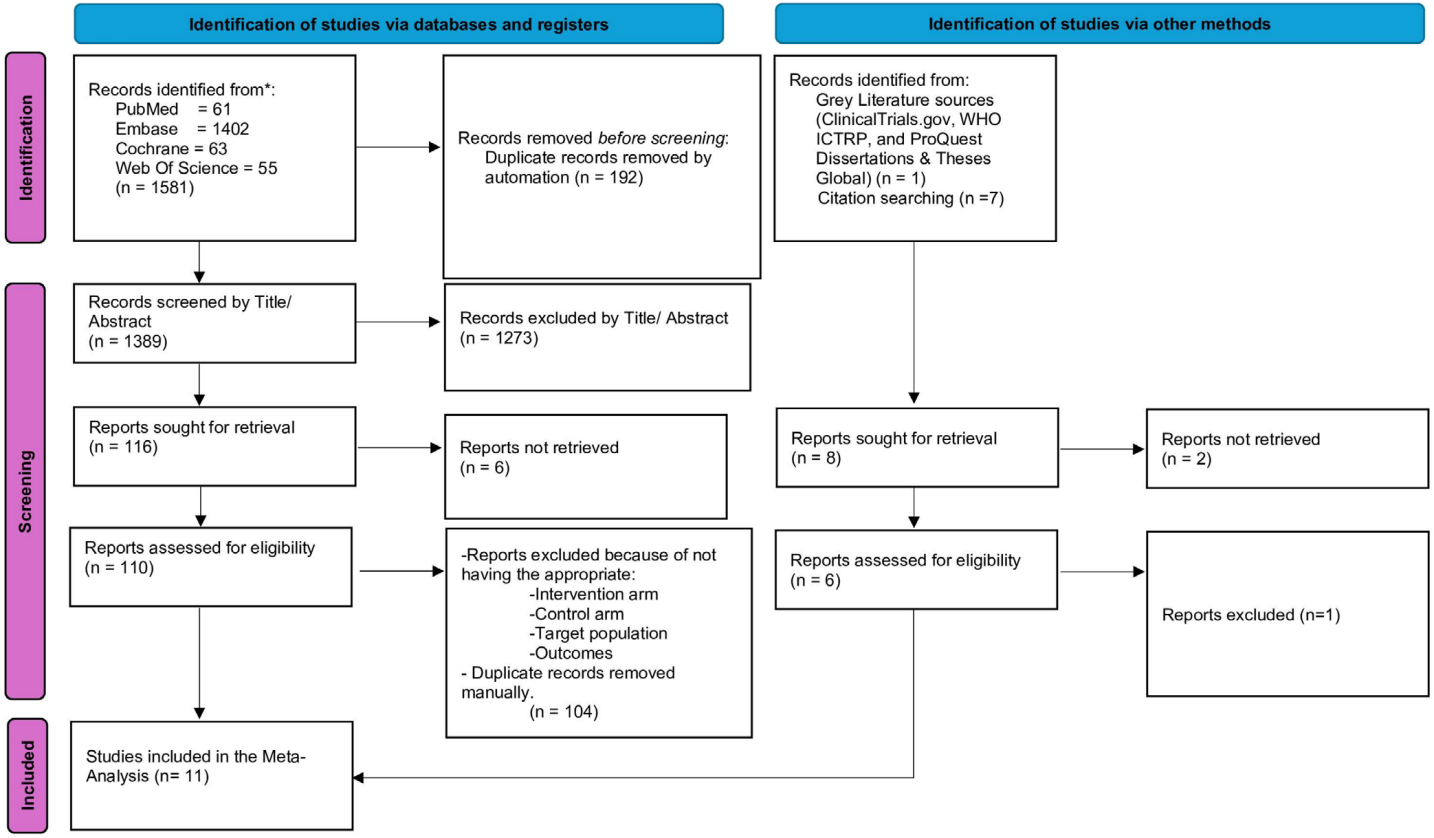
The Preferred Reporting Items for Systematic reviews and Meta‐Analyses (PRISMA) flow diagram showing the literature search and selection process of the included randomized controlled trials (RCTs) evaluating linaclotide plus polyethylene glycol (PEG) versus PEG alone for bowel preparation. WHO ICTRP, World Health Organization International Clinical Trials Registry Platform.

Out of a total of 1581 records identified from PubMed, EMBASE, CENTRAL, and Web of Science databases, and additional records identified through grey literature and citation searching (*n* = 8), 11 studies [15, 16, 17, 18, 19, 20, 21, 22, 23, 24, 25] were included in the final meta‐analysis after a rigorous screening process as outlined in the PRISMA flowchart. The final analysis comprised data from 11 trials, with patient numbers varying across different outcomes. The study selection process is depicted in the PRISMA flowchart (Figure 1).

All the included studies were RCTs conducted in China. Among them, eight and three studies used 3‐L and 2‐L PEG solutions, respectively, as the control. In terms of linaclotide dosage, two studies administered an 870‐μg dose [19, 25], three studies used a 290‐μg dose [15, 21, 22], and six studies employed a 580‐μg dose [16, 17, 18, 20, 23, 24]. In addition, one study [16] also employed a 290‐μg dose. The average age of the participants across these studies was 47.82 ± 18.4 years. The characteristics of the studies are detailed in Table 1.

**TABLE 1:** Baseline characteristics of patients included in the studies, detailing age, gender distribution, and study‐specific variables.

First author (year of publication)	Country	Study design	Total participants (*N*)	Intervention	Intervention group (*n*)	Control	Control group (*n*)	Age, years (mean ± SD)		BMI, kg/m^2^ (mean ± SD)		DM (*n*/*N*)		Constipation (*n*/*N*)	
								Intervention	Control	Intervention	Control	Intervention	Control	Intervention	Control
Wang (2023) [19]	China	RCT	130	Linaclotide (870 μg) + PEG (3 L); Linaclotide (870 μg) + PEG (2 L)	87 Male: 45 Female: 42	PEG (3 L)	43 Male: 23 Female: 20	NM	NM	22.56 ± 2.69	22.9 ± 3.3	NM	NM	NM	NM
Qi (2022) [25]	China	RCT	116	Linaclotide (870 μg) + PEG (3 L)	58 Male: 27 Female: 31	PEG (3 L)	58 Male: 30 Female: 28	58.10 ± 13.26	57.72 ± 13.87	NM	NM	6/58	9/58	NM	NM
Zhao (2021) [22]	China	RCT	315	Linaclotide (290 μg) + PEG (3 L)	105 Male: 29 Female: 76	PEG (3 L)	210 Male: 96 Female: 114	50 ± 11	50.0 ± 12.1	22.8 ± 2.75	22.7 ± 3.0	NM	NM	55/105	74/210
Cheng (2022) [15]	China	RCT	414	Linaclotide (290 μg) + PEG (3 L); Linaclotide (290 μg) + PEG (2 L)	262 Male: 142 Female: 120	PEG (3 L)	152 Male: 71 Female: 81	42.4 ± 12.3	45.2 ± 12.5	NM	NM	45/262	20/152	174/262	99/152
Yang (2023) [20]	China	RCT	154	Linaclotide (580 μg) + PEG (2 L)	142 Male: 67 Female: 75	PEG (2 L)	12 Male: 4 Female: 8	48.62 ± 13.27	50.83 ± 16.02	NM	NM	NM	NM	NM	NM
Tan (2022) [18]	China	RCT	240	Linaclotide (580 μg) + PEG (2 L)	117 of 120 Male: 63 Female: 54	PEG (3 L)	118 of 120 Male: 66 Female: 52	52.4 ± 7.3	53.9 ± 8.4	NM	NM	NM	NM	NM	NM
Zhang (2021) [21]	China	RCT	288	Linaclotide (290 μg) + PEG (2 L)	144 Male: 74 Female: 70	PEG (2 L)	144 Male: 80 Female: 64	50.7 ± 10.9	50.2 ± 10.9	24.3 ± 3.9	24.3 ± 3.2	18/144	16/144	34/144	31/144
Li (2023) [17]	China	RCT	304	Linaclotide (580 μg) + PEG (2 L)	152 Male: 79 Female: 73	PEG (3 L)	152 Male: 75 Female: 77	40.67 ± 10.37	39.3 ± 11.85	NM	NM	NM	NM	NM	NM
Zhang (2023) [23]	China	RCT	568	Linaclotide (580 μg) + PEG (1 L)	273 of 284 Male: 140 Female: 133	PEG (2 L)	275 of 284 Male: 156 Female: 119	45.08 ± 11.82	46.36 ± 11.53	22.43 ± 2.11	22.51 ± 2.13	NM	NM	NM	NM
Wu (2024) [24]	China	Multicenter, prospective RCT	470	Linaclotide (580 μg) + PEG (2 L)	231 of 235 Male: 170 Female: 61	PEG (3 L)	227 of 235 Male: 146 Female: 81	42.0 ± 12.5	42.0 ± 12.2	23.0 ± 3.3	23.5 ± 3.2	2/231	2/227	20/231	28/227
Liu (2024) [16]	China	Prospective, single‐blind RCT	753	Linaclotide (580 μg) + PEG (2 L); Linaclotide (290 μg) + PEG (2 L)	498 of 502 Male: 241 Female: 257	PEG (3 L)	251 Male: 127 Female: 124	50.85 ± 10.82	50.68 ± 12.01	23.2 ± 3.15	23.5 ± 3.3	33/498	20/251	84/498	56/251

Abbreviations: BMI, body mass index; NM, not mentioned; PEG, polyethylene glycol; RCT, randomized controlled trial; SD, standard deviation.

### 3.2 Risk of Bias and GRADE Assessment

**FIGURE 2:**
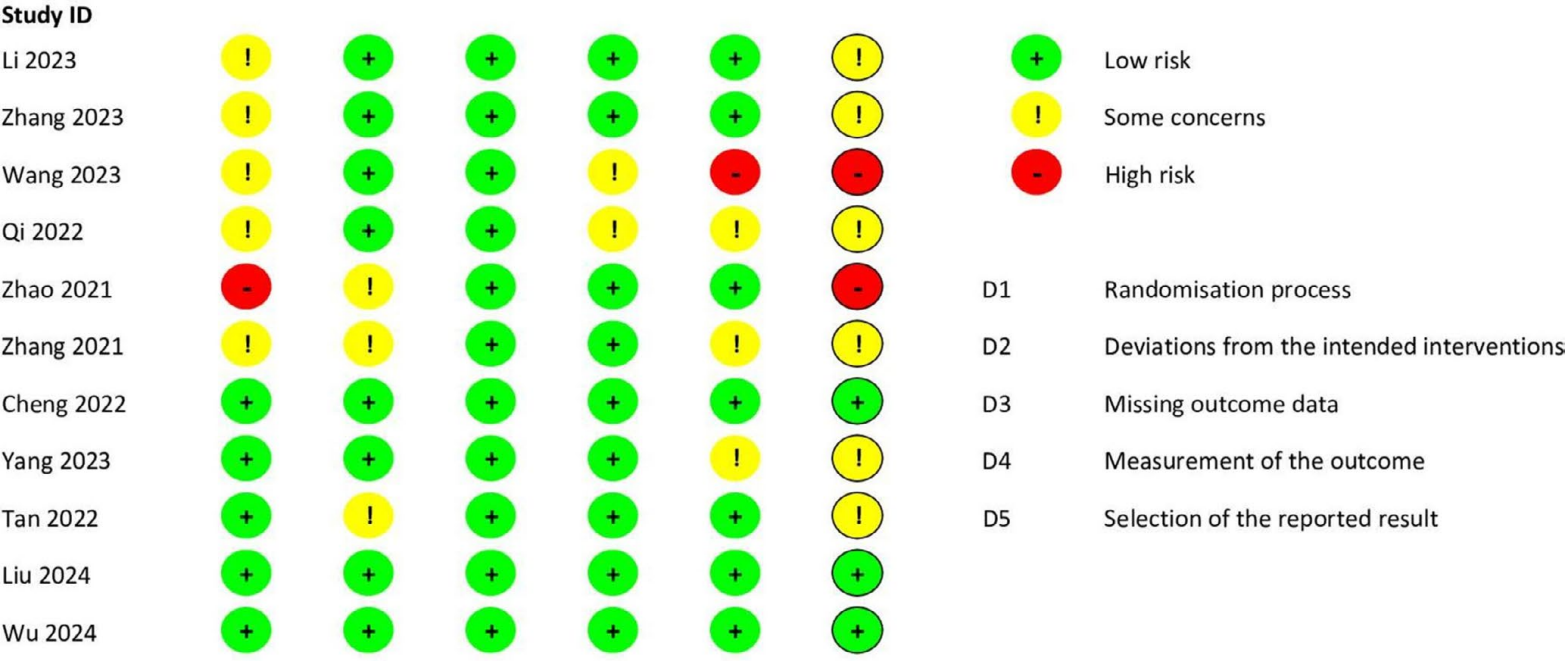
Risk of bias of the included randomized controlled trials (RCTs) using the Cochrane Risk of Bias Tool 2.0.

Among the included RCTs, three studies [15, 16, 24] were assessed as low risk, six studies [17, 18, 20, 21, 23, 25] had some concerns of bias, particularly due to issues in the randomization process and deviations from intended interventions, and the remaining two studies [19, 22] were classified as high risk (Figure 2). The certainty of evidence for each outcome assessed using the GRADE framework is available in Supporting Information.

### 3.3 Primary Outcomes

#### 3.3.1 Adequate Bowel Preparation

**FIGURE 3:**
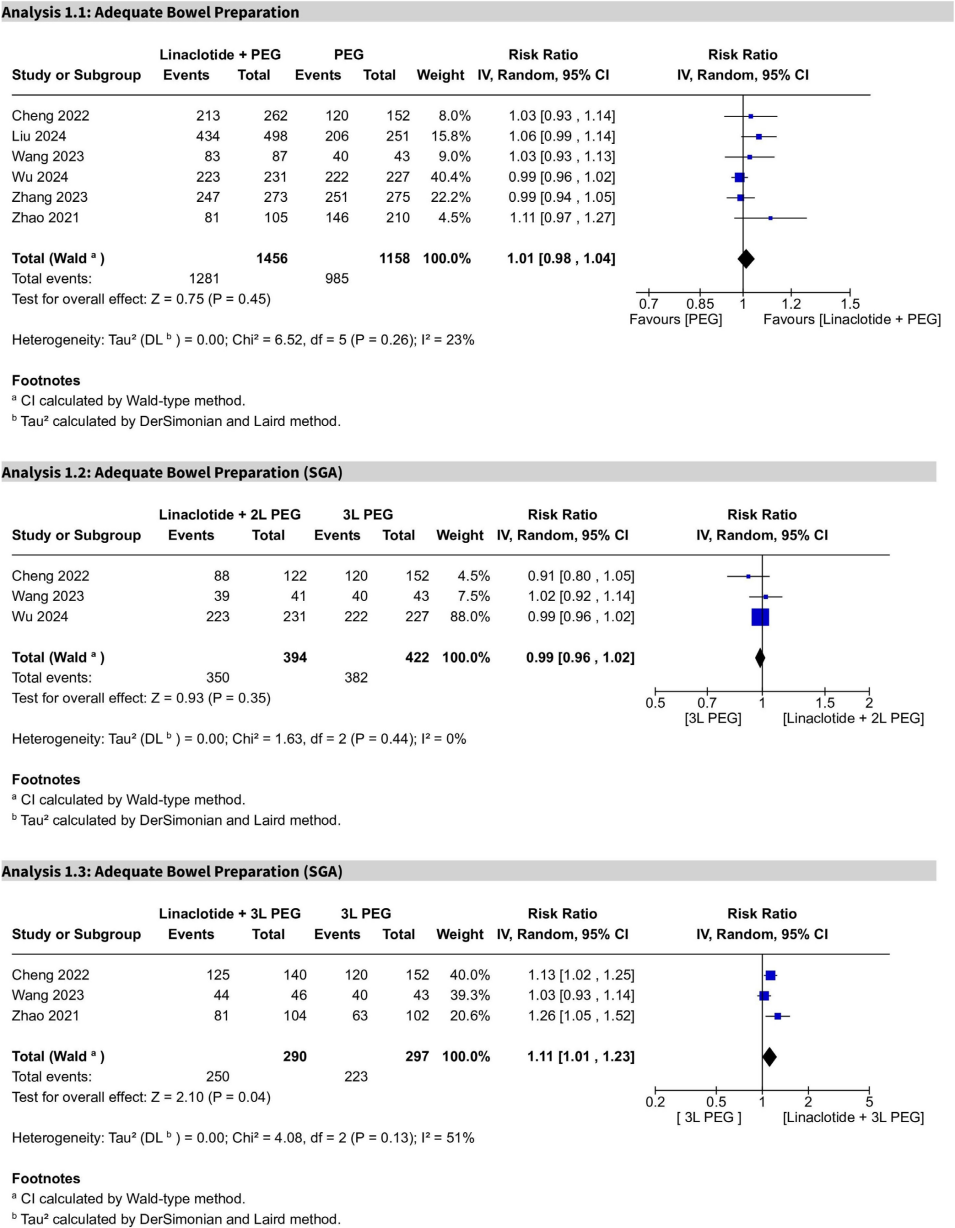
Forest plots of adequate bowel preparation comparing linaclotide plus polyethylene glycol (PEG) with PEG alone. Subgroup analyses are shown for (Analysis 1.1) overall comparison, (Analysis 1.2) 2‐L PEG plus linaclotide versus 3‐L PEG alone, and (Analysis 1.3) 3‐L PEG plus linaclotide versus 3‐L PEG alone.

Overall, the RR for adequate bowel preparation showed no statistical significance (RR 1.01, 95% CI 0.98–1.04, *p* = 0.45), indicating no clear difference between the PEG plus linaclotide and the PEG alone groups. Statistical heterogeneity was low (*I* ^2^ = 23%), and the certainty of evidence was rated as moderate (Figure 3). Sensitivity analysis led to the exclusion of Zhang et al.'s study [21] (Figure S1).

Subgroup analysis comparing 2‐L PEG plus linaclotide with 3‐L PEG alone revealed no statistically significant difference (RR 0.99, 95% CI 0.96–1.02, *p* = 0.35), with no heterogeneity (*I* ^2^ = 0%) (Figure 3). Sensitivity analysis resulted in the exclusion of Liu et al.'s study [16] (Figure S2).

For the comparison of 3‐L PEG plus linaclotide with 3‐L PEG alone, the RR for adequate bowel preparation was statistically significant (RR 1.11, 95% CI 1.01–1.23, *p* = 0.04), favoring the linaclotide plus 3‐L PEG group. Moderate heterogeneity was observed (*I* ^2^ = 51%) (Figure 3).

#### 3.3.2 BBPS Score

##### 3.3.2.1 Total BBPS Score

**FIGURE 4:**
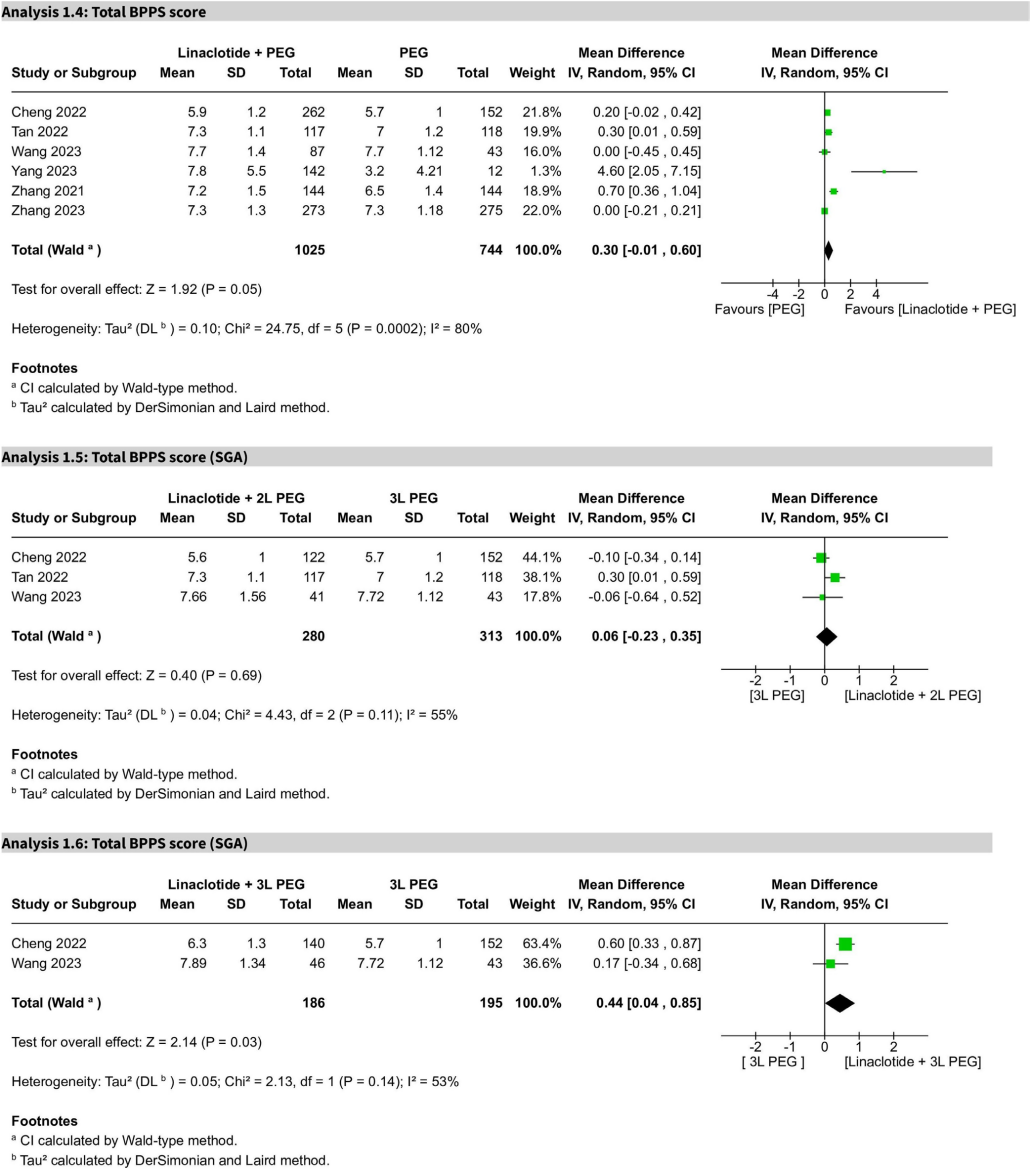
Forest plots of total Boston Bowel Preparation Scale (BBPS) scores comparing linaclotide plus polyethylene glycol (PEG) with PEG alone for bowel preparation. Subgroup analyses are shown for (Analysis 1.4) overall comparison, (Analysis 1.5) 2‐L PEG plus linaclotide versus 3‐L PEG, and (Analysis 1.6) 3‐L PEG plus linaclotide versus 3‐L PEG alone.

The overall MD for total BBPS score did not show any statistically significant difference (MD 0.43, 95% CI −0.05 to 0.91, *p* = 0.08), while the heterogeneity was significantly high (*I* ^2^ = 96%; Figure S3). Sensitivity analysis, involving step‐by‐step exclusion of the studies of Li et al. [17] and Qi et al. [25] (Figures S4 and S5), resulted in a revised MD of 0.30 (95% CI −0.01 to 0.60, *p* = 0.05), which continued to show no statistically significant difference with the *I* ^2^ value reduced to 80% (Figure 4). The certainty of evidence was rated as moderate.

For the subgroup comparison of 2‐L PEG plus linaclotide with 3‐L PEG alone, the MD for total BBPS score was not statistically significant (MD 0.06, 95% CI −0.23 to 0.35, *p* = 0.69), with moderate heterogeneity (*I* ^2^ = 55%) (Figure 4), following the exclusion of Li et al.'s study [17] by sensitivity analysis (Figure S6). While 3‐L PEG plus linaclotide in comparison with 3‐L PEG alone showed a statistically significant MD of 0.44 (95% CI 0.04–0.85, *p* = 0.03), favoring linaclotide, with moderate heterogeneity (*I* ^2^ = 53%) (Figure 4), following the exclusion of Qi et al.'s study [25] by sensitivity analysis (Figure S7).

##### 3.3.2.2 Left Colon BBPS Score

**FIGURE 5:**
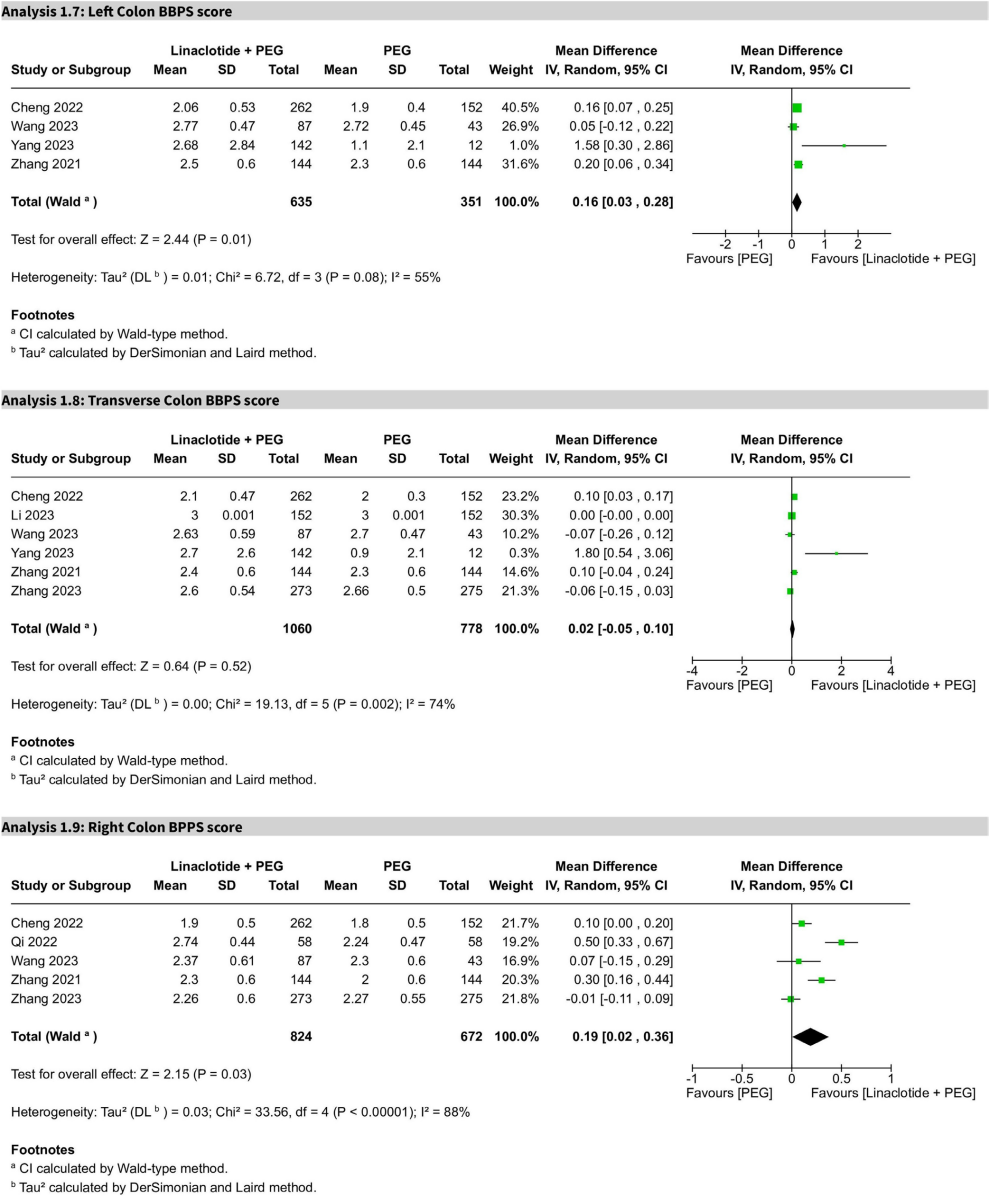
Forest plots of segmental Boston Bowel Preparation Scale (BBPS) scores comparing linaclotide plus polyethylene glycol (PEG) with PEG alone for (Analysis 1.7) left colon, (Analysis 1.8) transverse colon, and (Analysis 1.9) right colon.

Analysis of the left colon BBPS score, based on five trials, indicated statistical significance between the two groups (MD 0.23, 95% CI 0.08–0.38, *p* = 0.003), with a high heterogeneity (*I* ^2^ = 75%) (Figure S8). Sensitivity analysis, after the removal of Qi et al.'s study [25], showed that the MD for the left colon BBPS score remained statistically significant (MD 0.16, 95% CI 0.03–0.28, *p* = 0.01), favoring the linaclotide plus PEG group, with moderate heterogeneity (*I* ^2^ = 55%) (Figure 5). The certainty of evidence was also rated as moderate.

##### 3.3.2.3 Transverse Colon BBPS Score

In six trials, after excluding Qi et al.'s study [25] by sensitivity analysis (Figure S8), the MD for the transverse colon BBPS score was not statistically significant (MD 0.02, 95% CI −0.05 to 0.10, *p* = 0.52), with a high heterogeneity (*I* ^2^ = 74%) (Figure 5). The certainty of evidence was rated as moderate.

##### 3.3.2.4 Right Colon BBPS Score

Across five trials, the MD for the right colon BBPS score was statistically significant (MD 0.19, 95% CI 0.02–0.36, *p* = 0.03), favoring PEG plus linaclotide over PEG alone (Figure 5). Substantial heterogeneity was noted (*I* ^2^ = 88%), and the certainty of evidence was rated as low due to concerns regarding bias and imprecision.

#### 3.3.3 Impact on Timing Parameters of Colonoscopy

##### 3.3.3.1 Cecal Intubation Time

**FIGURE 6:**
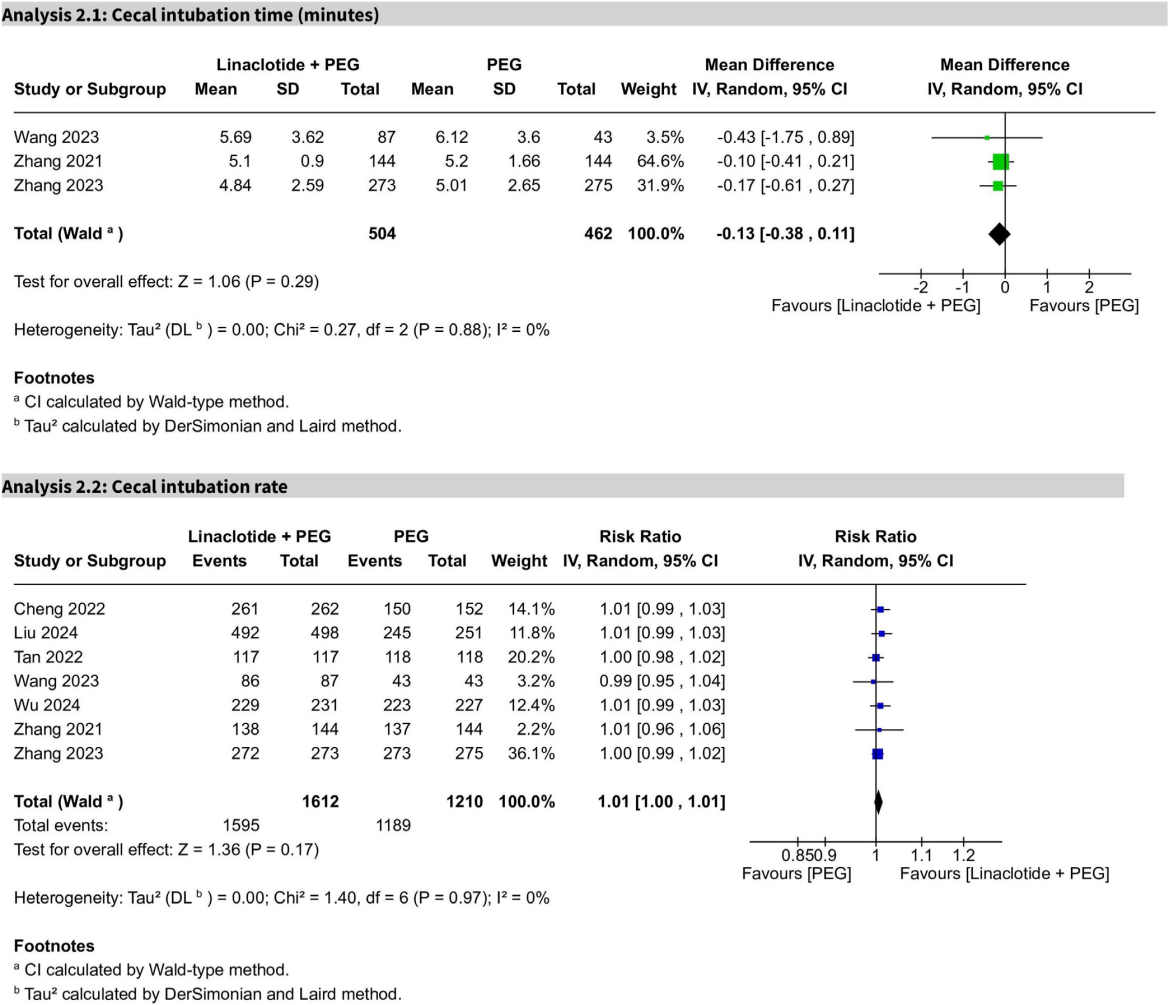
Forest plots for colonoscopy timing outcomes, (Analysis 2.1) cecal intubation time and (Analysis 2.2) cecal intubation rate, comparing linaclotide plus polyethylene glycol (PEG) with PEG alone for bowel preparation.

The MD for cecal intubation time, based on three trials, was not statistically significant (MD −0.13, 95% CI −0.38 to 0.11, *p* = 0.29), suggesting that addition of linaclotide did not significantly affect the cecal intubation time compared with PEG alone. No heterogeneity was observed (*I* ^2^ = 0%), and the certainty of evidence was rated as high (Figure 6).

##### 3.3.3.2 Cecal Intubation Rate

In seven trials, the cecal intubation rate showed no statistical significance (RR 1.01, 95% CI 1.00–1.01, *p* = 0.17), with no heterogeneity (*I* ^2^ = 0%) and a high certainty of evidence (Figure 6).

### 3.4 Secondary Outcomes

#### 3.4.1 PDR

**FIGURE 7:**
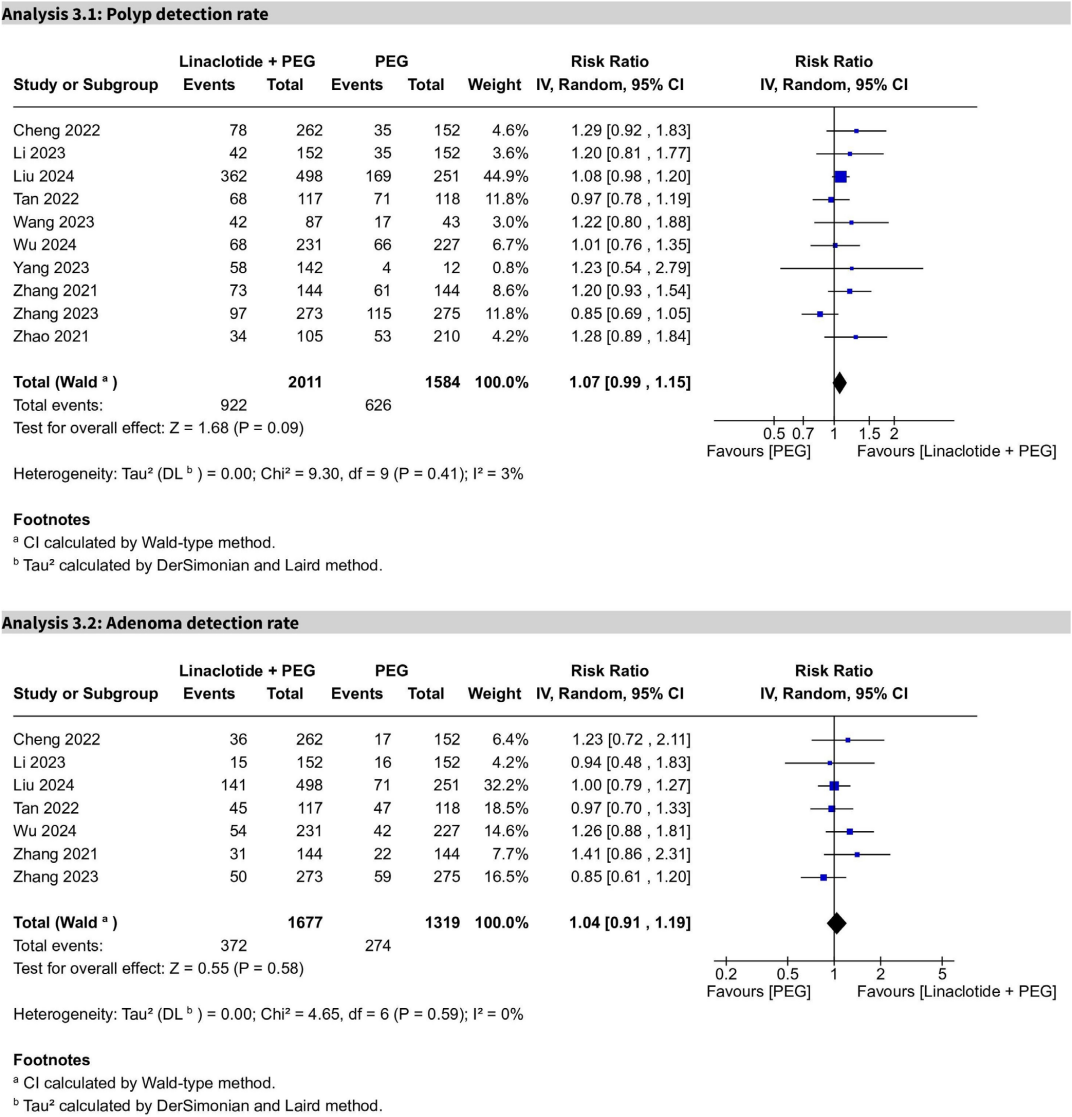
Forest plots comparing linaclotide plus polyethylene glycol (PEG) with PEG alone for (Analysis 2.1) polyp detection rate and (Analysis 2.2) adenoma detection rate.

In 10 trials, the PDR showed no statistical significance (RR 1.07, 95% CI 0.99–1.15, *p* = 0.09). Low heterogeneity was observed (*I* ^2^ = 3%), and the certainty of evidence was rated as high (Figure 7).

In subgroup analysis comparing 2‐L PEG plus linaclotide with 3‐L PEG alone, there was no statistically significant difference (RR 1.07, 95% CI 0.99–1.17, *p* = 0.10), with no heterogeneity (*I* ^2^ = 0%) (Figure S9). In addition, after excluding Wang et al.'s study [19] via the sensitivity analysis (Figure S10), the 3‐L PEG plus linaclotide showed statistical significance when compared to 3‐L PEG alone (RR 1.78, 95% CI 1.32–2.40, *p* = 0.0001), with no heterogeneity (*I* ^2^ = 0%), which favored the linaclotide plus PEG group (Figure S11).

#### 3.4.2 ADR

In seven trials, the ADR was not statistically significant (RR 1.04, 95% CI 0.91–1.19, *p* = 0.58), indicating no difference between the PEG plus linaclotide and the PEG alone groups. Heterogeneity was low (*I* ^2^ = 0%), and the certainty of evidence was moderate (Figure 7).

#### 3.4.3 Adverse Events

##### 3.4.3.1 Abdominal Pain

**FIGURE 8:**
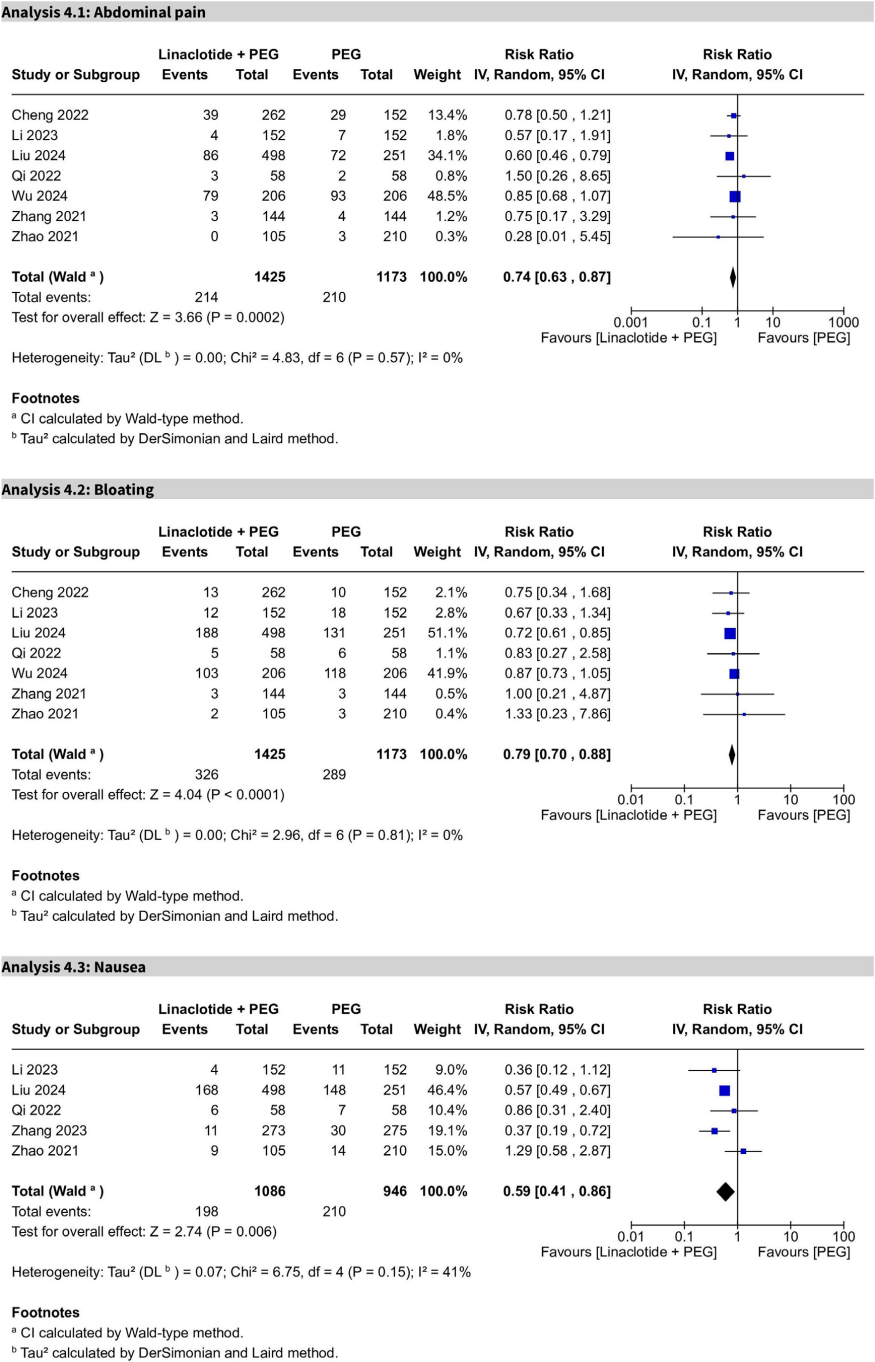
Forest plots of adverse events comparing linaclotide plus polyethylene glycol (PEG) with PEG alone for (Analysis 4.1) abdominal pain, (Analysis 4.2) abdominal bloating, and (Analysis 4.3) nausea.

Reported in seven trials, abdominal pain was significantly lower in the PEG plus linaclotide group (RR 0.74, 95% CI 0.63–0.87, *p* = 0.0002), with no heterogeneity (*I* ^2^ = 0%). The certainty of evidence was rated as high (Figure 8).

##### 3.4.3.2 Abdominal Bloating

Abdominal bloating across seven trials was significantly low in the PEG plus linaclotide group (RR 0.79, 95% CI 0.70–0.88, *p* < 0.0001). There was no heterogeneity (*I* ^2^ = 0%), and the certainty of evidence was rated as high (Figure 8).

##### 3.4.3.3 Nausea

Nausea, reported in five trials, demonstrated statistical significance, with a lower incidence in the PEG plus linaclotide group (RR 0.59, 95% CI 0.41–0.86, *p* = 0.006) (Figure 8). Moderate heterogeneity was noted (*I* ^2^ = 41%), after excluding Zhang et al.'s study [21]. The certainty of evidence was rated as high (Figure S12).

##### 3.4.3.4 Sleep Disturbance

**FIGURE 9:**
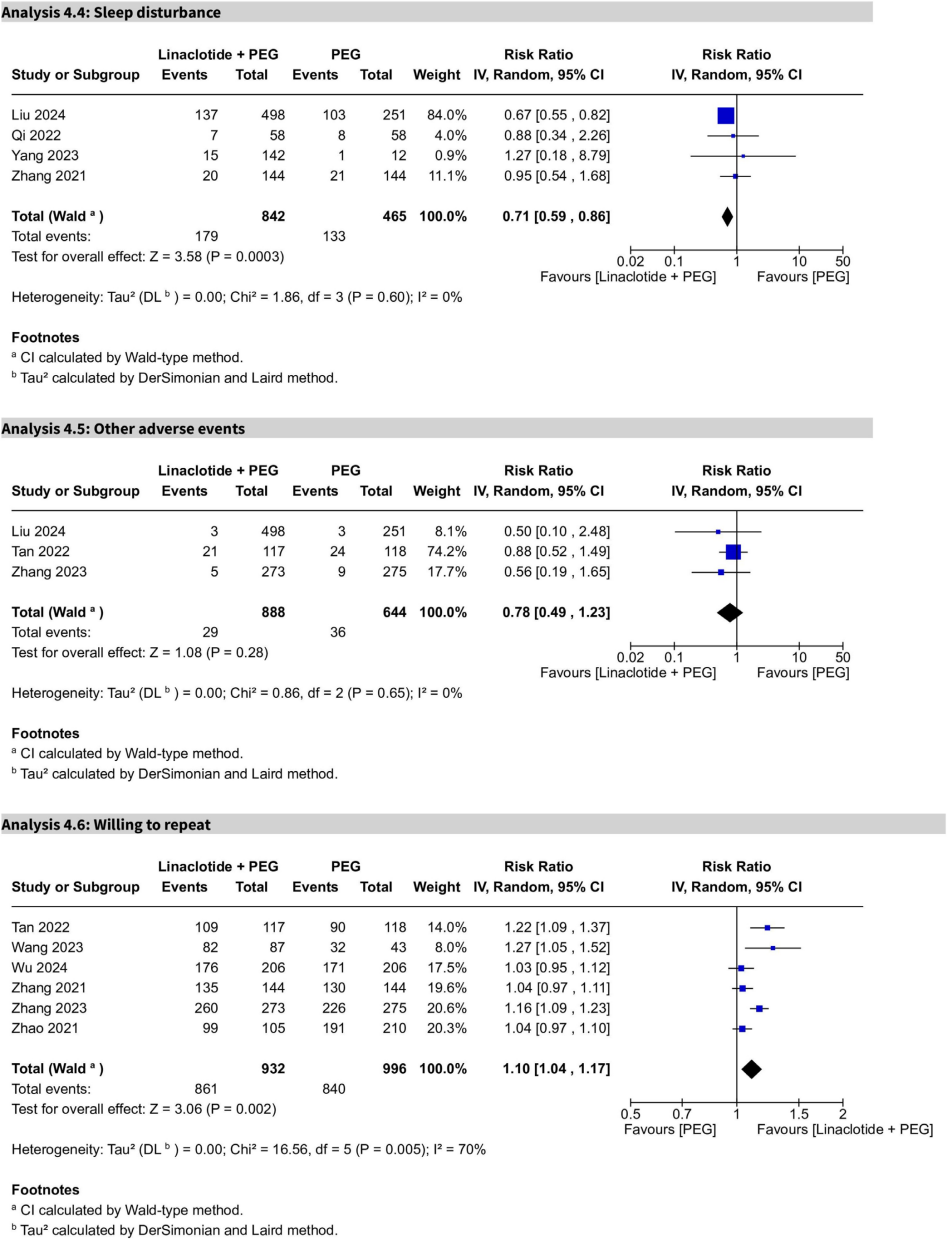
Forest plots comparing linaclotide plus polyethylene glycol (PEG) with PEG alone for (Analysis 4.4) sleep disturbance, (Analysis 4.5) other adverse events, and (Analysis 4.6) willingness to repeat the colonoscopic procedure.

Sleep disturbance, evaluated across four trials, showed statistical significance, with a lower incidence in the PEG plus linaclotide group (RR 0.71, 95% CI 0.59–0.86, *p* = 0.0003) (Figure 9). No heterogeneity was observed (*I* ^2^ = 0%) after excluding Zhang et al.'s study [23] via the sensitivity analysis (Figure S12), and the certainty of evidence was rated as high.

##### 3.4.3.5 Other Adverse Events

Other adverse events, reported in three trials, showed no statistical significance (RR 0.78, 95% CI 0.49–1.23, *p* = 0.28), indicating that linaclotide plus PEG did not differ significantly from the PEG alone group in terms of other safety profile (Figure 9). No heterogeneity was observed (*I* ^2^ = 0%), and the certainty of evidence was rated low.

#### 3.4.4 Willingness to Repeat the Colonoscopic Procedure

Assessed in six trials, willingness to repeat the colonoscopic procedure was statistically significant in favor of the addition of linaclotide (RR 1.10, 95% CI 1.04–1.17, *p* = 0.002) (Figure 9), after excluding Liu et al.'s study via sensitivity analysis [16] (Figure S13). High heterogeneity was noted (*I* ^2^ = 70%), and the certainty of evidence was rated as moderate.

#### 3.4.5 Withdrawal Time

Across seven trials, the withdrawal time was not statistically significant between the two groups (MD −0.03, 95% CI −0.23 to 0.17, *p* = 0.78), with a high heterogeneity (*I* ^2^ = 67%) (Figure S14). The sensitivity analysis excluded Qi et al.'s study [25] (Figure S15). The certainty of evidence was rated as low.

After excluding Wang et al.'s study [19] via sensitivity analysis (Figure S16), subgroup analysis for withdrawal time comparing 2‐L PEG plus linaclotide with 3‐L PEG showed no significant difference (MD 0.02, 95% CI −0.17 to 0.20, *p* = 0.86), with moderate heterogeneity (*I* ^2^ = 43%) (Figure S17).

Similarly, the 3‐L PEG plus linaclotide, when compared with 3‐L PEG alone, also showed no statistical significance (MD −0.52, 95% CI −1.31 to 0.26, *p* = 0.19), with a high heterogeneity (*I* ^2^ = 77%), after excluding Qi et al.'s study [25] (Figures S18 and S19).

## 4 Discussion

Linaclotide has proven to be an important therapeutic advancement for the treatment of IBS and chronic constipation by stimulating secretion of chloride and bicarbonate into the intestinal lumen via activation of the CFTR ion channel, resulting in increased intestinal fluid secretion and accelerated GI transit [26]. It is, however, imperative to explore its potential role in bowel preparation protocols. Our findings indicate that while the addition of linaclotide to PEG is comparable to PEG alone with respect to overall bowel preparation quality, total and transverse colon BBPS score, cecal intubation rate and time, withdrawal time, and ADR and PDR, several notable advantages are observed. Specifically, the linaclotide plus PEG group showed significantly higher right and left colon BBPS scores, reduced incidences of adverse events—including abdominal pain, nausea, abdominal bloating, and sleep disturbance—as well as a higher willingness to repeat the procedure, suggesting improved patient tolerability and satisfaction. Subgroup analyses regarding adequate bowel preparation further revealed that the combination of 2‐L PEG with linaclotide was non‐inferior to the 3‐L PEG regimen, while the 3‐L PEG plus linaclotide regimen showed superiority over the 3‐L PEG alone regimen.

Optimal colonoscopy outcomes depend heavily on adequate bowel preparation which ensures a clear endoscopic field. Although our analysis did not consistently show statistically significant benefits of linaclotide plus PEG versus PEG alone in all primary end‐points, the linaclotide plus PEG group achieved higher right and left colon BBPS scores. These findings align with prior studies by Zhang et al. [23] and Yang et al. [20], which also reported comparable efficacy between linaclotide‐based regimens and standard PEG protocols. However, variations in dosing and timing of bowel preparation across studies may influence these outcomes. To address this heterogeneity, analyses were stratified by doses of the regimens. The results indicated that the 3‐L PEG plus linaclotide regimen was superior to 3‐L PEG alone to achieve adequate bowel preparation, possibly due to the ability of linaclotide to enhance colonic motility. This stratified analysis also highlighted regional variations, as 3‐L PEG is commonly employed in China [27], whereas 4‐L PEG remains the standard in the United States [28]. The 2‐L PEG plus linaclotide regimen demonstrated non‐inferiority to the 3‐L PEG regimen, suggesting potential reductions in required PEG volumes while maintaining efficacy.

BBPS, which is a standardized tool for evaluating bowel preparation quality through segmental scores, revealed significantly higher total scores in the linaclotide plus PEG group compared to the PEG alone group in the current study, aligning with findings by Cheng et al. [15]. Notably, the left colon demonstrated statistically significant superior cleansing efficacy in the linaclotide group. This finding suggests particular benefit in improving visualization for patients with prior sigmoid interventions or those undergoing early repeat surveillance colonoscopies. Prior research investigating the purgative effects of linaclotide for capsule endoscopy and colonoscopy has reported minimal adverse effects, which are consistent with our findings [8, 21]. Our analysis showed that adverse events such as abdominal bloating, pain, nausea, and sleep disturbance were significantly lower in the linaclotide plus PEG group compared to the PEG alone group, underscoring its favorable safety profile and potential to enhance patient compliance and satisfaction. Unlike PEG, which often causes intolerance due to high‐volume consumption and unpleasant taste [28, 29], linaclotide reduces liquid intake requirements and offers a cost‐effective alternative [23]. The increased willingness to repeat the procedure in patients receiving linaclotide underscores its tolerability and potential patient‐centered benefits.

Adequate bowel preparation is crucial for procedural success and optimizing ADR, inversely correlating with CRC incidence. Insufficient bowel preparation can result in incomplete cecal visualization, increasing the risk of interval CRC [30]. Nevertheless, our study did not reveal significant differences in cecal intubation rate or time, PDR, ADR, or withdrawal time between linaclotide plus PEG and PEG alone. These outcomes suggest that while linaclotide does not compromise procedural efficiency, its primary benefits are related to improved patient tolerability and experience. These findings are in contrast to those of Qi et al., who reported reduced entry and exit time in the linaclotide group, possibly reflecting better bowel preparation quality and leading to shorter procedure time [25]. Additionally, subgroup analysis revealed significant improvements in PDR for the 3‐L PEG plus linaclotide group versus 3‐L PEG alone, potentially attributed to enhanced mucosal visualization facilitated by superior bowel cleansing, as indicated by higher BBPS scores.

The strengths of our study include synthesizing data from 11 studies, thus providing robust analytical power. Stratified analyses offered valuable comparative insights into bowel preparation regimens across regional practices in China, and consistent outcome measures further enhanced data uniformity and interpretability. Compared with the recent network meta‐analysis by Elgendy et al. [31], we restricted inclusion to 1–3 L PEG regimens in average‐risk patients and excluded those with chronic constipation, which reduced clinical heterogeneity for the adequacy outcome (*I* ^2^ = 23% vs. 83%) and yielded results that are directly applicable to contemporary low‐volume protocols. Moreover, the pairwise methodological framework circumvents the transitivity assumptions inherent to network models, allowing clear, volume‐specific effect estimates for 2‐L and 3‐L bowel preparation regimens.

Despite these strengths, several limitations should be acknowledged. Subgroup analyses revealed varying levels of heterogeneity across outcome measures, which might have hindered interpretive consistency. The exclusive use of the BBPS instead of the Ottawa Bowel Preparation Scale could have introduced interpretative bias. Moreover, patient‐reported outcomes such as willingness to repeat the procedure are inherently subjective and may be influenced by recall bias. Variability in linaclotide dosages and administration timing across the included studies might have also influenced the results. Owing to this heterogeneity, we were unable to conduct a meaningful subgroup analysis based on linaclotide dosage. Additionally, the majority of study participants were low‐risk individuals with minimal comorbidities, and all trials were conducted in China, limiting the generalizability of our findings. Future research should aim to address these limitations to better define the role of linaclotide in clinical practice.

## 5 Conclusions

In conclusion, linaclotide shows promise as an adjunct to PEG in bowel preparation protocols by enhancing patient tolerability, increasing segmental BBPS scores, and potentially reducing PEG volumes required, without compromising preparation quality or procedural efficiency. Future studies should aim at standardizing linaclotide dosage and its timing of administration and evaluating its efficacy across more diverse patient populations to better define its clinical application.

## Conflicts of Interest

The authors declare no conflicts of interest.

## Supporting information


**Table S1:** GRADE for individual outcomes.

**Figure S1:** Sensitivity analysis for adequate bowel preparation before the exclusion of Zhang et al.’s study [21]. CI, confidence interval; PEG, polyethylene glycol.

**Figure S2:** Sensitivity analysis for adequate bowel preparation (2‐L polyethylene glycol [PEG] plus linaclotide vs. 3‐L PEG) before the exclusion of Liu et al.’ study [16]. CI, confidence interval.

**Figure S3:** Forest plot of total Boston Bowel Preparation Scale (BBPS) score (overall comparison, prior to any exclusions). CI, confidence interval.

**Figure S4:** Sensitivity analysis for total Boston Bowel Preparation Scale (BBPS) score after exclusion of Li et al.’s study. [17]. CI, confidence interval.

**Figure S5:** Sensitivity analysis for total Boston Bowel Preparation Scale (BBPS) score after exclusion of Qi et al.’s study. [25]. CI, confidence interval.

**Figure S6:** Sensitivity analysis for total Boston Bowel Preparation Scale (BBPS) score (2‐L polyethylene glycol [PEG] plus linaclotide vs. 3‐L PEG) before exclusion of Li et al.’s study [17]. CI, confidence interval.

**Figure S7:** Sensitivity analysis for total Boston Bowel Preparation Scale (BBPS) score (3‐L polyethylene glycol [PEG] plus linaclotide vs. 3‐L PEG) before exclusion of Qi et al.’s study [25]. CI, confidence interval.

**Figure S8:** Sensitivity analysis for segmental Boston Bowel Preparation Scale (BBPS) scores for (Analysis 1.7) left and (Analysis 1.8) transverse colon before the exclusion of Qi et al.’s study [25]. CI, confidence interval.

**Figure S9:** Subgroup analysis of polyp detection rate (2‐L polyethylene glycol [PEG] plus linaclotide vs. 3‐L PEG). CI, confidence interval.

**Figure S10:** Subgroup analysis of polyp detection rate (3‐L polyethylene glycol [PEG] plus linaclotide vs. 3‐L PEG) before the exclusion of Wang et al.’s study [19]. CI, confidence interval.

**Figure S11:** Sensitivity analysis for polyp detection rate (3‐L polyethylene glycol [PEG] plus linaclotide vs. 3‐L PEG) before exclusion of Wang et al.’s study [19]. CI, confidence interval.

**Figure S12:** Sensitivity analyses for (Analysis 4.3) nausea and (Analysis 4.4) sleep disturbance before exclusion of Zhang et al.’s studies [21, 23]. CI, confidence interval.

**Figure S13:** Sensitivity analysis for willingness to repeat the colonoscopy procedure before exclusion of Liu et al.’ study [16]. CI, confidence interval.

**Figure S14:** Forest plot of withdrawal time comparing linaclotide plus polyethylene glycol [PEG] versus PEG alone for bowel preparation. CI, confidence interval.

**Figure S15:** Sensitivity analysis for withdrawal time comparing linaclotide plus polyethylene glycol [PEG] versus PEG alone for bowel preparation before exclusion of Qi et al.’s study [25]. CI, confidence interval.

**Figure S16:** Sensitivity analysis of withdrawal time (2‐L polyethylene glycol [PEG] plus linaclotide vs. 3‐L PEG) before exclusion of Wang et al.’s study [19]. CI, confidence interval.

**Figure S17:** Subgroup analysis of withdrawal time (2‐L polyethylene glycol [PEG] plus linaclotide vs. 3‐L PEG) after exclusion of Wang et al.’s study [19]. CI, confidence interval.

**Figure S18:** Subgroup analysis of withdrawal time (3‐L polyethylene glycol [PEG] plus linaclotide vs. 3‐L PEG) after exclusion of Qi et al.’s study [25]. CI, confidence interval.

**Figure S19:** Sensitivity analysis of withdrawal time (3‐L polyethylene glycol [PEG] plus linaclotide vs. 3‐L PEG) before exclusion of Qi et al.’s study [25]. CI, confidence interval.

## Data Availability

The data that support the findings of this study are available from the corresponding author upon reasonable request.
