# Supplementary material for: Efficacy and Safety of Linaclotide as an Adjunct to Polyethylene Glycol in Bowel Preparation: A Meta‐Analysis
Source: J Dig Dis. 2025 Sep 16;26(7-8):318–33. doi: 10.1111/1751-2980.70008 (PMC12492060; doi:10.1111/1751-2980.70008)
Supplement: Supplementary file 1 — Table S1: GRADE for individual outcomes. [file CDD-26-318-s007.docx]

**Supplementary Information**

**Search strategy.**

For MEDLINE (via PubMed):

| 1 | ‘Linaclotide’ OR ‘ASP-0456’ OR ‘ASP0456’ OR ‘MD-1100’ OR ‘Linzess’ |
| --- | --- |
| 2 | ‘PEG’ OR ‘Goly*’ OR ‘Golytely’ OR ‘Polyethy*’ OR ‘Polyoxyethylenes’ OR ‘Polyoxyethylene’ OR ‘Polyglycol’ OR ‘Polyglycols’ OR ‘Glycol, Polyethylene’ OR ‘Glycols, Polyethylene’ |
| 3 | ‘Cathartics’ OR ‘Bowel Evacuants’ OR ‘Purgatives’ OR ‘Bowel Preparation Solutions’ OR ‘Bowel prep*’ |
| 4 | #1 AND #2 AND #3 |

For EMBASE (Elsevier):

| 1 | ‘Linaclotide’ OR ‘ASP-0456’ OR ‘ASP0456’ OR ‘MD-1100’ OR ‘Linzess’ |
| --- | --- |
| 2 | ‘PEG’ OR ‘Goly*’ OR ‘Golytely’ OR ‘Polyethy*’ OR ‘Polyoxyethylenes’ OR ‘Polyoxyethylene’ OR ‘Polyglycol’ OR ‘Polyglycols’ OR ‘Glycol, Polyethylene’ OR ‘Glycols, Polyethylene’ |
| 3 | ‘Cathartics’ OR ‘Bowel Evacuants’ OR ‘Purgatives’ OR ‘Bowel Preparation Solutions’ OR ‘Bowel prep*’ |
| 4 | #1 AND #2 AND #3 |

For Cochrane Central Register of Controlled Trials:

| 1 | ‘Linaclotide’ OR ‘ASP-0456’ OR ‘ASP0456’ OR ‘MD-1100’ OR ‘Linzess’ |
| --- | --- |
| 2 | ‘PEG’ OR ‘Goly*’ OR ‘Golytely’ OR ‘Polyethy*’ OR ‘Polyoxyethylenes’ OR ‘Polyoxyethylene’ OR ‘Polyglycol’ OR ‘Polyglycols’ OR ‘Glycol, Polyethylene’ OR ‘Glycols, Polyethylene’ |
| 3 | ‘Cathartics’ OR ‘Bowel Evacuants’ OR ‘Purgatives’ OR ‘Bowel Preparation Solutions’ OR ‘Bowel prep*’ |
| 4 | #1 AND #2 AND #3 |

For Web of Science:

| 1 | ‘Linaclotide’ OR ‘ASP-0456’ OR ‘ASP0456’ OR ‘MD-1100’ OR ‘Linzess’ |
| --- | --- |
| 2 | ‘PEG’ OR ‘Goly*’ OR ‘Golytely’ OR ‘Polyethy*’ OR ‘Polyoxyethylenes’ OR ‘Polyoxyethylene’ OR ‘Polyglycol’ OR ‘Polyglycols’ OR ‘Glycol, Polyethylene’ OR ‘Glycols, Polyethylene’ |
| 3 | ‘Cathartics’ OR ‘Bowel Evacuants’ OR ‘Purgatives’ OR ‘Bowel Preparation Solutions’ OR ‘Bowel prep*’ |
| 4 | #1 AND #2 AND #3 |

For Grey Literatures:

| Sources | Search Strategies | Notes/Filters |
| --- | --- | --- |
| ClinicalTrials.gov | ("Linaclotide" OR "ASP-0456" OR "ASP0456" OR "MD-1100" OR "Linzess") AND ("Polyethylene glycol" OR "PEG" OR "Golytely" OR "Goly*") AND ("Bowel preparation" OR "Cathartics" OR "Purgatives" OR "Bowel prep*") | The search was performed with Condition or disease set to *Colonoscopy* and Other terms including *Linaclotide* and *PEG*. |
| WHO ICTRP | ("Linaclotide" OR "Linzess" OR "ASP-0456" OR "MD-1100") AND ("Polyethylene glycol" OR "PEG" OR "Golytely") AND ("Bowel preparation" OR "Bowel prep*") | Multiple simplified searches were run, for example "Linaclotide AND PEG" and "Linzess AND Bowel preparation". |
| ProQuest Dissertations & Theses | ("Linaclotide" OR "Linzess" OR "ASP-0456" OR "MD-1100") AND ("Polyethylene glycol" OR "PEG" OR "Polyethy*" OR "Golytely" OR "Goly*") AND ("Bowel preparation" OR "Bowel prep*" OR "Cathartics" OR "Purgatives") | Searches were filtered to Dissertations & Theses only, with subject area restricted to *Medicine / Health Sciences*. |

**Supplementary Table 1. GRADE for individual outcomes**

| **Certainty assessment** | | | | | | | **№ of patients** | | **Effect** | | **Certainty** | **Importance** |
| --- | --- | --- | --- | --- | --- | --- | --- | --- | --- | --- | --- | --- |
| **№ of studies** | **Study design** | **Risk of bias** | **Inconsistency** | **Indirectness** | **Imprecision** | **Other considerations** | **Bowel Preparation Assessment** | **Placebo** | **Relative (95% CI)** | **Absolute (95% CI)** |  |  |
| **Adequate Bowel Preparation** | | | | | | | | | | | | |
| 6 | Randomized Trials | Not Serious | Not Serious | Not Serious | Serious | None | 1281/1456 (88.0%) | 985/1158 (85.1%) | **RR 1.01** (0.98 to 1.04) | **9 more per 1000** (from 17 fewer to 34 more) | ⨁⨁⨁◯ Moderate |  |
| **Total BPPS score** | | | | | | | | | | | | |
| 6 | Randomized trials | Serious | Not serious | Not serious | Not serious | None | 1025 | 744 | - | MD **0.3 higher** (0.01 lower to 0.6 higher) | ⨁⨁⨁◯ Moderate |  |
| **Left Colon BBPS score** | | | | | | | | | | | | |
| 4 | Randomized trials | Not serious | Not serious | Not serious | Serious | None | 635 | 351 | - | MD **0.16 higher** (0.03 higher to 0.28 higher) | ⨁⨁⨁◯ Moderate |  |
| **Transverse Colon BBPS score** | | | | | | | | | | | | |
| 6 | Randomized trials | Not serious | Not serious | Not serious | Serious | None | 1060 | 778 | - | MD **0.02 higher** (0.05 lower to 0.10 higher) | ⨁⨁⨁◯ Moderate |  |
| **Right Colon BPPS score** | | | | | | | | | | | | |
| 5 | Randomized trials | Serious | Not serious | Not serious | Serious | None | 824 | 672 | - | MD **0.19 higher** (0.02 higher to 0.36 higher) | ⨁⨁◯◯ Low |  |
| **Cecal intubation time (minutes)** | | | | | | | | | | | | |
| 3 | Randomized Trials | Not Serious | Not Serious | Not Serious | Serious | None | **504** | **462** | **-** | **MD 0.13 lower (0.38 lower to 0.11 higher)** | ⨁⨁⨁⨁ High |  |
| **Cecal intubation rate** | | | | | | | | | | | | |
| 7 | Randomized Trials | Not Serious | Not Serious | Not Serious | Serious | None | **1595/1612 (98.9%)** | **1189/1210 (98.3%)** | **RR 1.01 (1.00 to 1.01)** | **10 more per 1,000 (from 0 fewer to 10 more)** | ⨁⨁⨁⨁ High |  |
| **Polyp detection rate** | | | | | | | | | | | | |
| 10 | Randomized Trials | Not Serious | Not Serious | Not Serious | Not Serious | None | **922/2011 (45.8%)** | **626/1584 (39.5%)** | **RR 1.07 (0.99 to 1.15)** | **28 more per 1,000 (from 4 fewer to 59 more)** | ⨁⨁⨁⨁ High |  |
| **Adenoma detection rate** | | | | | | | | | | | | |
| 7 | Randomized Trials | Not Serious | Not Serious | Not Serious | Not Serious | None | **372/1677 (22.2%)** | **274/1319 (20.8%)** | **RR 1.04 (0.91 to 1.19)** | **8 more per 1,000 (from 19 fewer to 39 more)** | ⨁⨁⨁◯ Moderate |  |
| **Abdominal pain** | | | | | | | | | | | | |
| 7 | Randomized Trials | Not Serious | Not Serious | Not Serious | Not Serious | None | **214/1425 (15.0%)** | **210/1173 (17.9%)** | **RR 0.74 (0.63 to 0.87)** | **47 fewer per 1,000 (from 66 fewer to 23 fewer)** | ⨁⨁⨁⨁ High |  |
| **Bloating** | | | | | | | | | | | | |
| 7 | Randomized Trials | Not Serious | Not Serious | Not Serious | Not Serious | None | **326/1425 (22.9%)** | **289/1173 (24.6%)** | **RR 0.79 (0.70 to 0.88)** | **52 fewer per 1,000 (from 74 fewer to 30 fewer)** | ⨁⨁⨁⨁ High |  |
| **Nausea** | | | | | | | | | | | | |
| 5 | Randomized Trials | Not Serious | Not Serious | Not Serious | Not Serious | None | **198/1086 (18.2%)** | **210/946 (22.2%)** | **RR 0.59 (0.41 to 0.86)** | **91 fewer per 1,000 (from 131 fewer to 31 fewer)** | ⨁⨁⨁⨁ High |  |
| **Sleep disturbance** | | | | | | | | | | | | |
| 4 | Randomized Trials | Not Serious | Not Serious | Not Serious | Not Serious | None | **179/842 (21.3%)** | **133/465 (28.6%)** | **RR 0.71 (0.59 to 0.86)** | **83 fewer per 1,000 (from 117 fewer to 40 fewer)** | ⨁⨁⨁⨁ High |  |
| **Other adverse events** | | | | | | | | | | | | |
| 3 | Randomized Trials | Serious | Not Serious | Not Serious | Serious | None | **29/888 (3.3%)** | **36/644 (5.6%)** | **RR 0.78 (0.49 to 1.23)** | **12 fewer per 1,000 (from 29 fewer to 13 more)** | ⨁⨁◯◯ Low |  |
| **Willing to repeat the colonoscopy procedure** | | | | | | | | | | | | |
| 4 | Randomized Trials | Not Serious | Serious | Serious | Not Serious | None | **653/710 (92.0%)** | **559/668 (83.7%)** | **RR 1.10 (1.02 to 1.19)** | **84 more per 1,000 (from 17 more to 159 more)** | ⨁⨁⨁◯ Moderate |  |
| **Withdrawal time** | | | | | | | | | | | | |
| 7 | Randomized Trials | Serious | Not Serious | Not Serious | Serious | None | **1486** | **1193** | **-** | **MD 0.03 lower (0.23 lower to 0.17 higher)** | ⨁⨁◯◯ Low |  |

Abbreviations: CI, confidence interval; MD, mean difference; RR, risk ratio.

**Supplementary Figure Legends**

**Figure S1.** Sensitivity analysis for adequate bowel preparation before the exclusion of Zhang et al.’s study [21]. CI, confidence interval; PEG, polyethylene glycol.

**Figure S2.** Sensitivity analysis for adequate bowel preparation (2-L polyethylene glycol [PEG] plus linaclotide versus 3-L PEG) before exclusion of Liu et al.’s study [16]. CI, confidence interval.

**Figure S3.** Forest plot of total Boston Bowel Preparation Scale (BBPS) score (overall comparison, prior to any exclusions). CI, confidence interval.

**Figure S4.** Sensitivity analysis for total Boston Bowel Preparation Scale (BBPS) score after exclusion of Li et al.’s study [17]. CI, confidence interval.

**Figure S5.** Sensitivity analysis for total Boston Bowel Preparation Scale (BBPS) score after exclusion of Qi et al.’s study [25]. CI, confidence interval.

**Figure S6.** Sensitivity analysis for total Boston Bowel Preparation Scale (BBPS) score (2-L polyethylene glycol [PEG] plus linaclotide versus 3-L PEG) before exclusion of Li et al.’s study [17]. CI, confidence interval.

**Figure S7.** Sensitivity analysis for total Boston Bowel Preparation Scale (BBPS) score (3-L polyethylene glycol [PEG] plus linaclotide versus 3-L PEG) before exclusion of Qi et al.’s study [25]. CI, confidence interval.

**Figure S8.** Sensitivity analysis for segmental Boston Bowel Preparation Scale (BBPS) scores for (Analysis 1.7) left and (Analysis 1.8) transverse colon before the exclusion of Qi et al.’s study [25]. CI, confidence interval.

**Figure S9.** Subgroup analysis of polyp detection rate (2-L polyethylene glycol [PEG] plus linaclotide versus 3-L PEG). CI, confidence interval.

**Figure S10.** Subgroup analysis of polyp detection rate (3-L polyethylene glycol [PEG] plus linaclotide versus 3-L PEG) before exclusion of Wang et al.’s study [19]. CI, confidence interval.

**Figure S11.** Sensitivity analysis for polyp detection rate (3-L polyethylene glycol [PEG] plus linaclotide versus 3-L PEG) after exclusion of Wang et al.’s study [19]. CI, confidence interval.

**Figure S12.** Sensitivity analyses for (Analysis 4.3) nausea and (Analysis 4.4) sleep disturbance before exclusion of Zhang et al.’s studies [21, 23]. CI, confidence interval.

**Figure S13.** Sensitivity analysis for willingness to repeat the colonoscopy procedure comparing linaclotide plus polyethylene glycol (PEG) with PEG alone for bowel preparation before exclusion of Liu et al.’s study [16]. CI, confidence interval.

**Figure S14.** Forest plot of withdrawal time comparing linaclotide plus polyethylene glycol (PEG) with PEG alone for bowel preparation. CI, confidence interval.

**Figure S15.** Sensitivity analysis for withdrawal time comparing linaclotide plus polyethylene glycol (PEG) with PEG alone for bowel preparation before exclusion of Qi et al.’s study [25]. CI, confidence interval.

**Figure S16.** Sensitivity analysis of withdrawal time (2-L polyethylene glycol [PEG] plus linaclotide versus 3-L PEG) before exclusion of Wang et al.’s study [19]. CI, confidence interval.

**Figure S17.** Subgroup analysis of withdrawal time (2-L polyethylene glycol [PEG] plus linaclotide versus 3-L PEG) after exclusion of Wang et al.’s study [19]. CI, confidence interval.

**Figure S18.** Subgroup analysis of withdrawal time (3-L polyethylene glycol [PEG] plus linaclotide versus 3-L PEG) after exclusion of Qi et al.’s study [25]. CI, confidence interval.

**Figure S19.** Sensitivity analysis of withdrawal time (3-L polyethylene glycol [PEG] plus linaclotide versus 3-L PEG) before exclusion of Qi et al.’s study [25]. CI, confidence interval.
