# Supplementary figures and images for: Efficacy and Safety of Linaclotide as an Adjunct to Polyethylene Glycol in Bowel Preparation: A Meta‐Analysis
Source: J Dig Dis. 2025 Sep 16;26(7-8):318–33. doi: 10.1111/1751-2980.70008 (PMC12492060; doi:10.1111/1751-2980.70008)

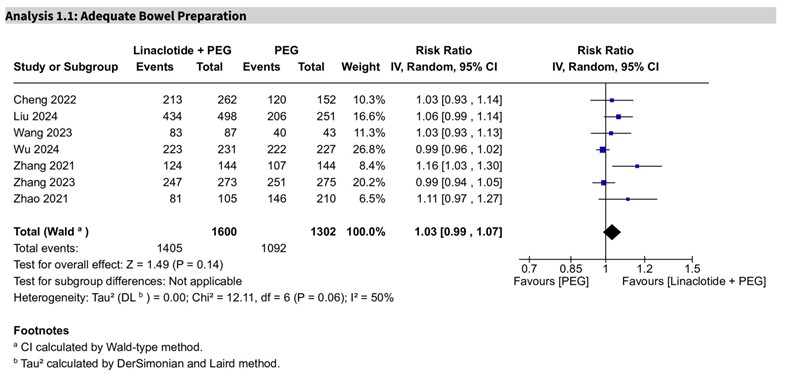

Supplement: Supplementary file 2 — Figure S1: Sensitivity analysis for adequate bowel preparation before the exclusion of Zhang et al.’s study [21]. CI, confidence interval; PEG, polyethylene glycol. [file CDD-26-318-s012.jpg]

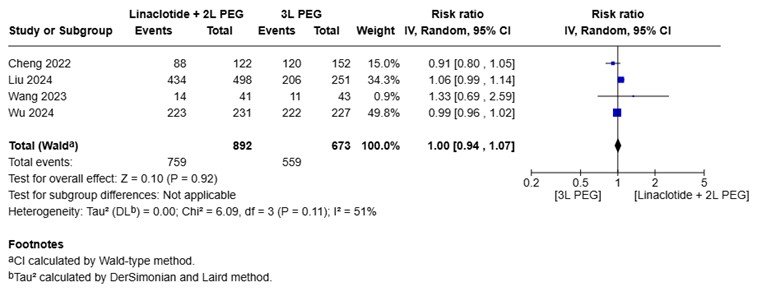

Supplement: Supplementary file 3 — Figure S2: Sensitivity analysis for adequate bowel preparation (2‐L polyethylene glycol [PEG] plus linaclotide vs. 3‐L PEG) before the exclusion of Liu et al.’ study [16]. CI, confidence interval. [file CDD-26-318-s006.jpg]

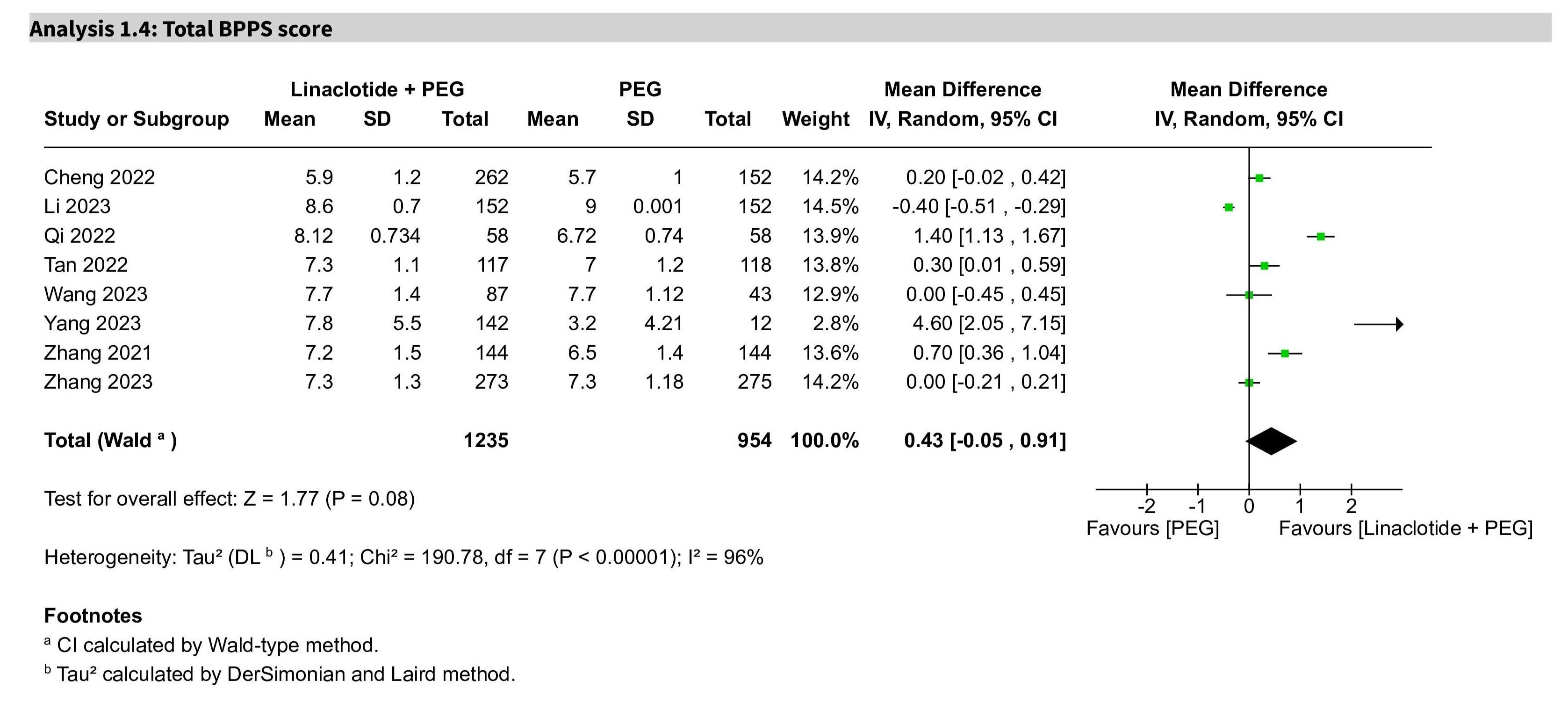

Supplement: Supplementary file 4 — Figure S3: Forest plot of total Boston Bowel Preparation Scale (BBPS) score (overall comparison, prior to any exclusions). CI, confidence interval. [file CDD-26-318-s003.jpg]

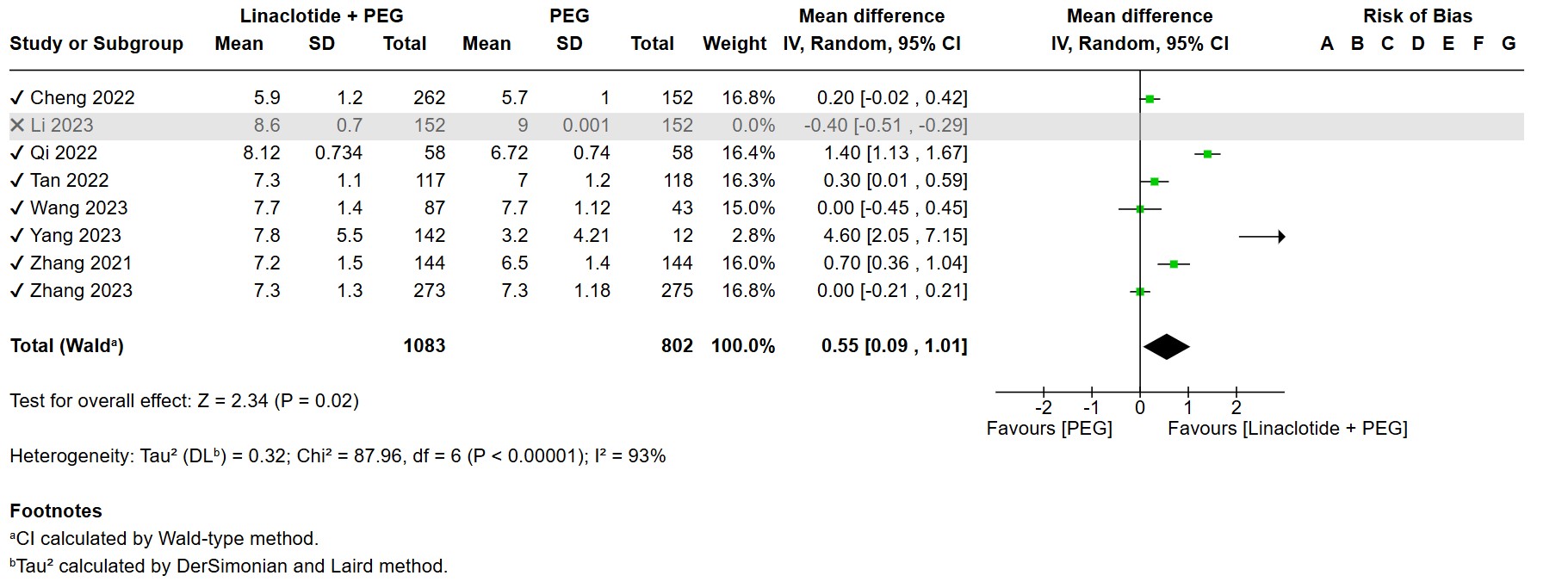

Supplement: Supplementary file 5 — Figure S4: Sensitivity analysis for total Boston Bowel Preparation Scale (BBPS) score after exclusion of Li et al.’s study. [17]. CI, confidence interval. [file CDD-26-318-s005.jpg]

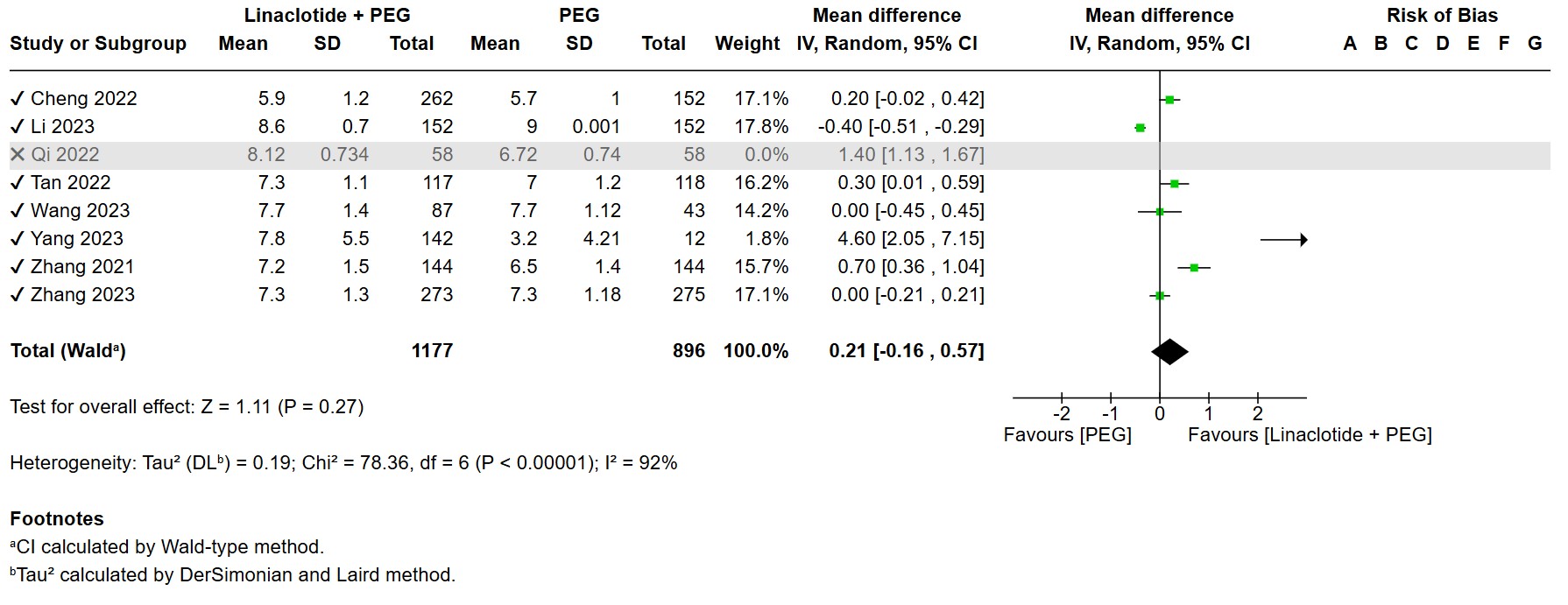

Supplement: Supplementary file 6 — Figure S5: Sensitivity analysis for total Boston Bowel Preparation Scale (BBPS) score after exclusion of Qi et al.’s study. [25]. CI, confidence interval. [file CDD-26-318-s010.jpg]

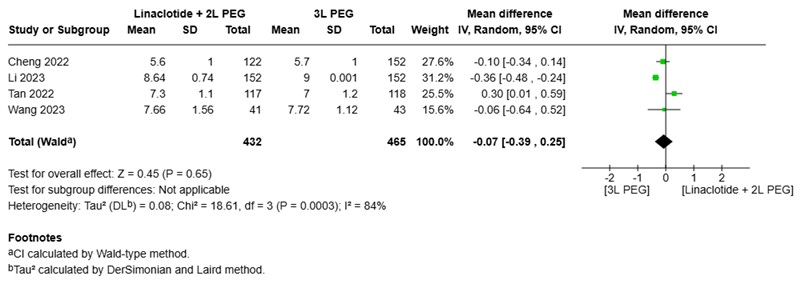

Supplement: Supplementary file 7 — Figure S6: Sensitivity analysis for total Boston Bowel Preparation Scale (BBPS) score (2‐L polyethylene glycol [PEG] plus linaclotide vs. 3‐L PEG) before exclusion of Li et al.’s study [17]. CI, confidence interval. [file CDD-26-318-s015.jpg]

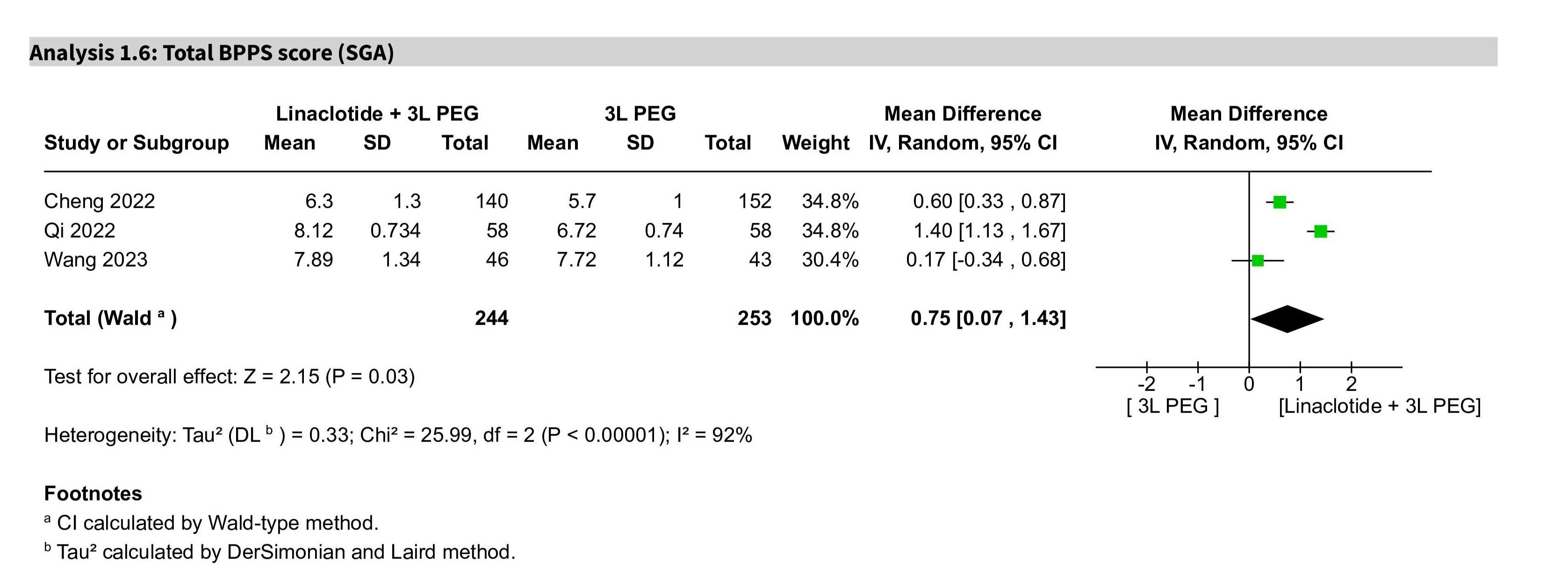

Supplement: Supplementary file 8 — Figure S7: Sensitivity analysis for total Boston Bowel Preparation Scale (BBPS) score (3‐L polyethylene glycol [PEG] plus linaclotide vs. 3‐L PEG) before exclusion of Qi et al.’s study [25]. CI, confidence interval. [file CDD-26-318-s016.jpg]

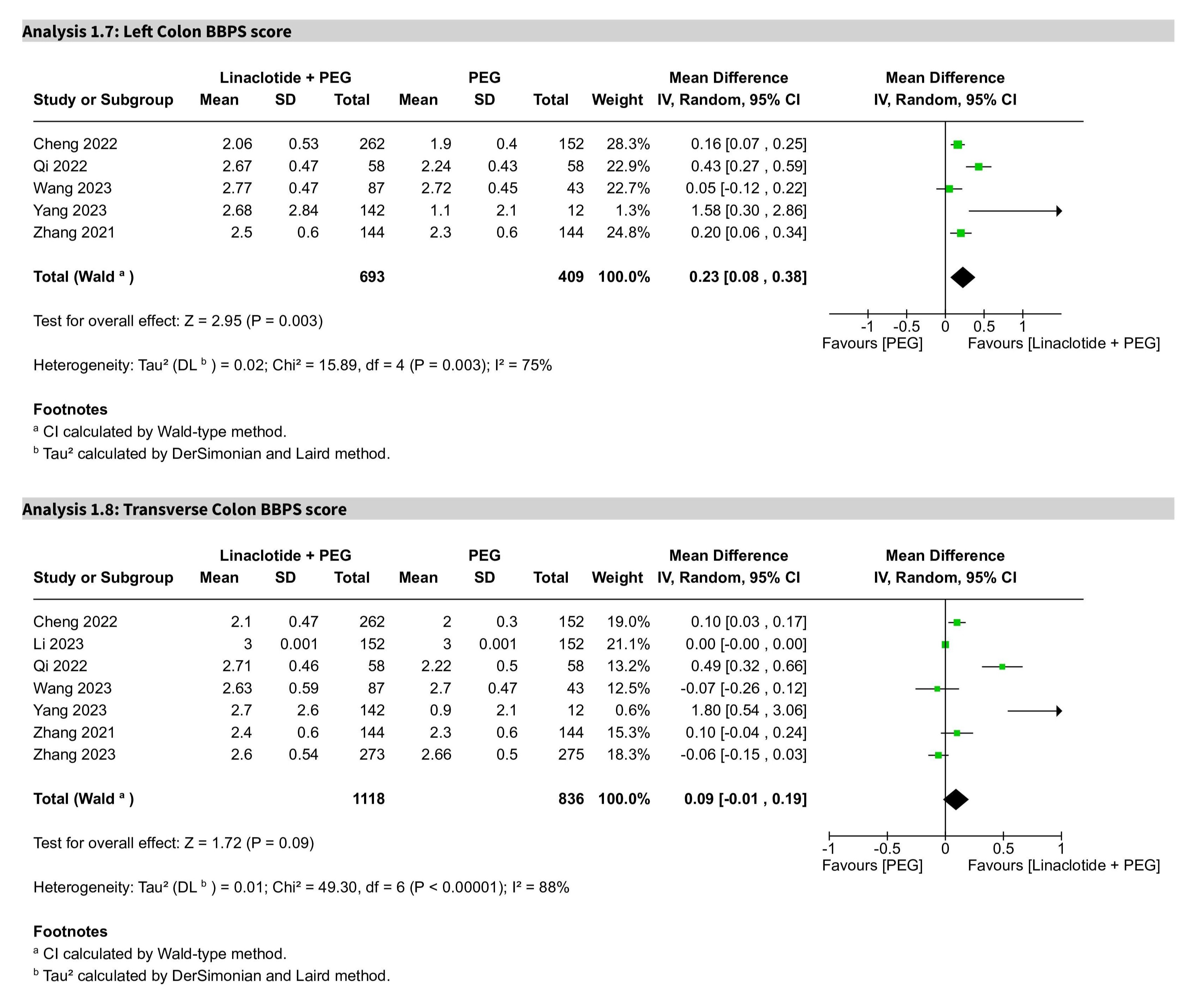

Supplement: Supplementary file 9 — Figure S8: Sensitivity analysis for segmental Boston Bowel Preparation Scale (BBPS) scores for (Analysis 1.7) left and (Analysis 1.8) transverse colon before the exclusion of Qi et al.’s study [25]. CI, confidence interval. [file CDD-26-318-s017.jpg]

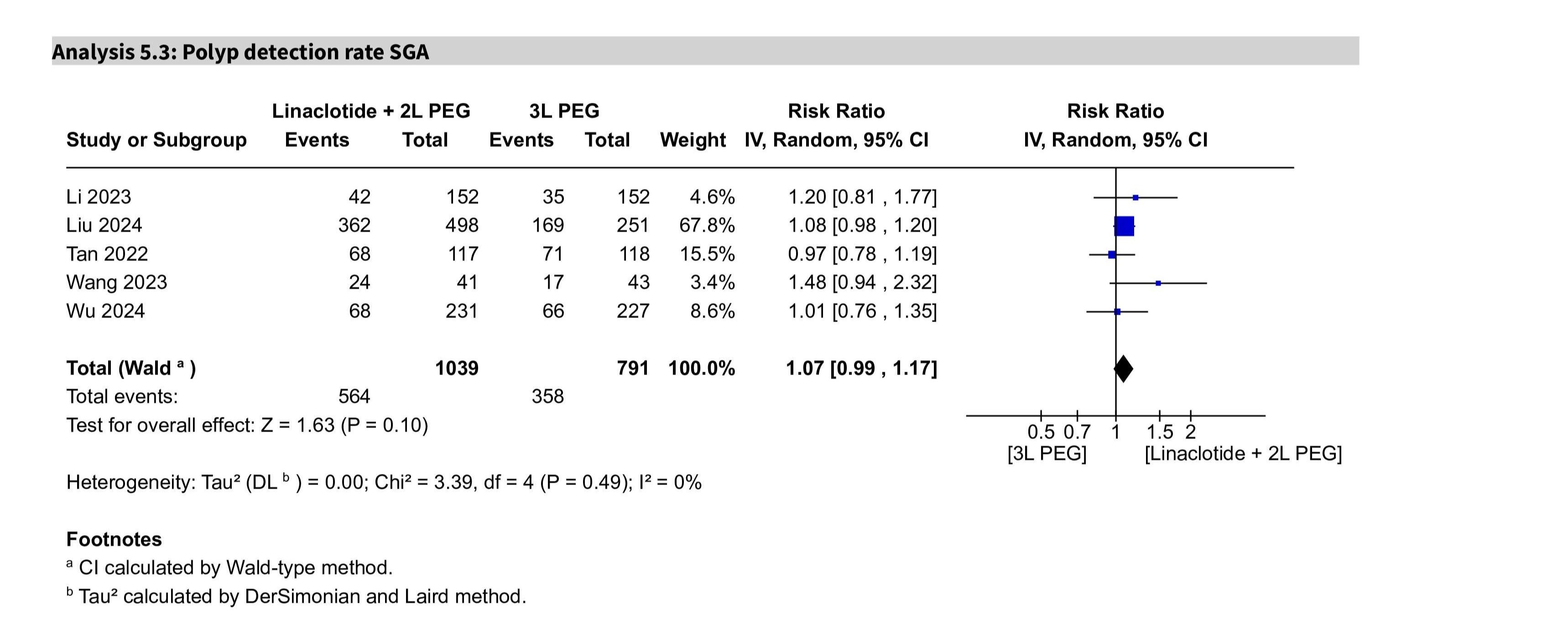

Supplement: Supplementary file 10 — Figure S9: Subgroup analysis of polyp detection rate (2‐L polyethylene glycol [PEG] plus linaclotide vs. 3‐L PEG). CI, confidence interval. [file CDD-26-318-s019.jpg]

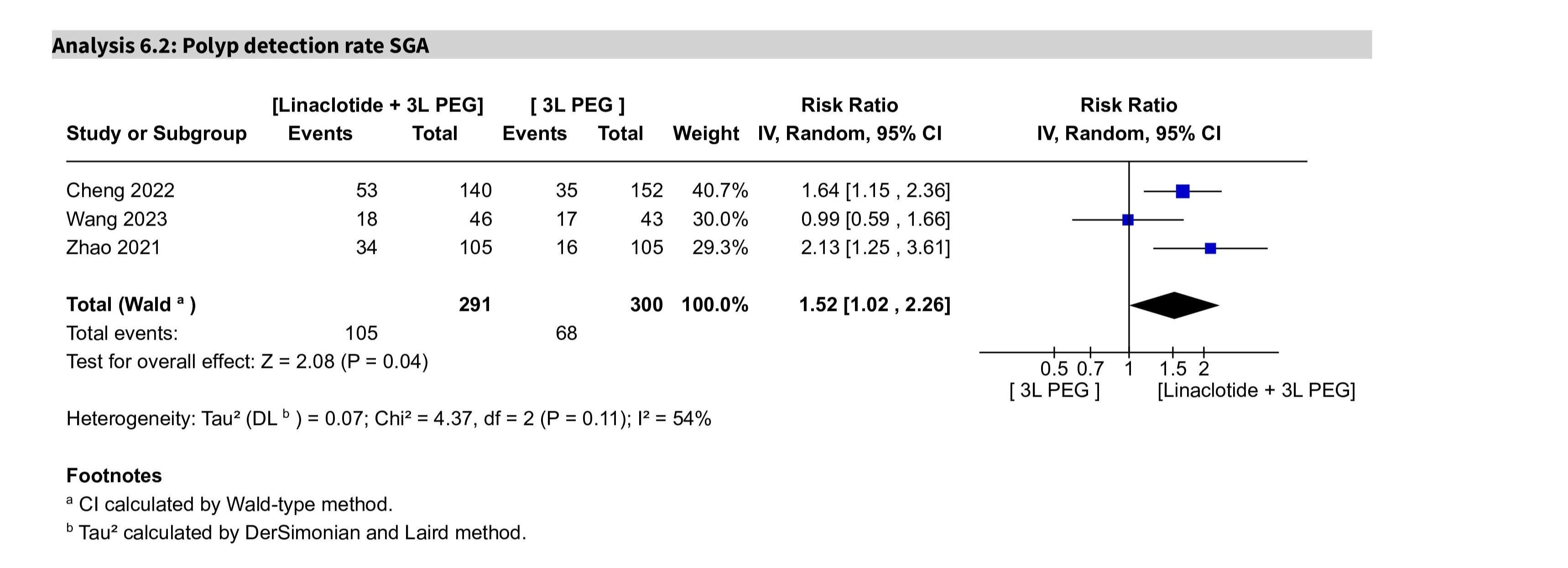

Supplement: Supplementary file 11 — Figure S10: Subgroup analysis of polyp detection rate (3‐L polyethylene glycol [PEG] plus linaclotide vs. 3‐L PEG) before the exclusion of Wang et al.’s study [19]. CI, confidence interval. [file CDD-26-318-s002.jpg]

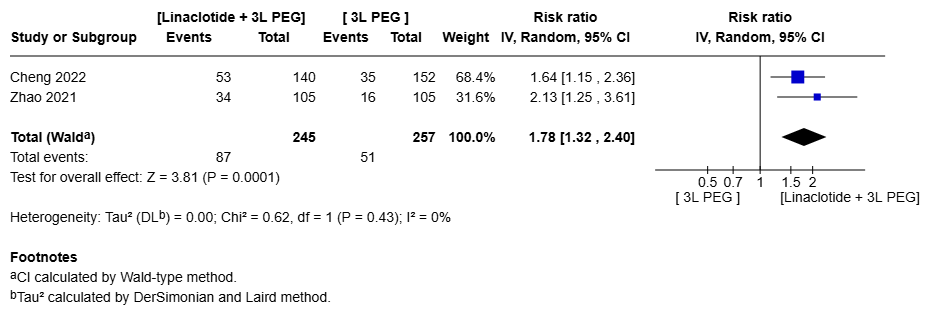

Supplement: Supplementary file 12 — Figure S11: Sensitivity analysis for polyp detection rate (3‐L polyethylene glycol [PEG] plus linaclotide vs. 3‐L PEG) before exclusion of Wang et al.’s study [19]. CI, confidence interval. [file CDD-26-318-s020.png]

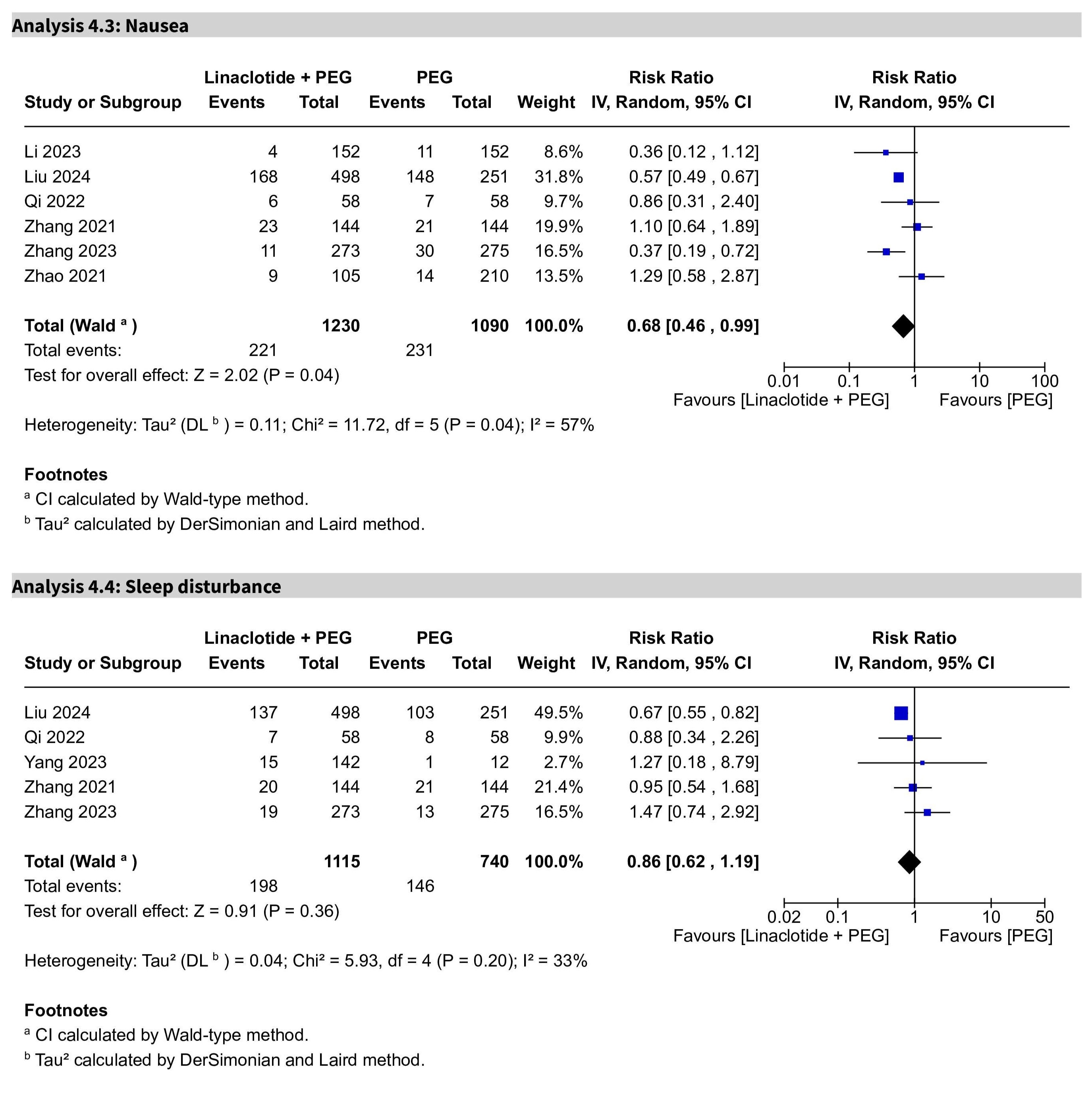

Supplement: Supplementary file 13 — Figure S12: Sensitivity analyses for (Analysis 4.3) nausea and (Analysis 4.4) sleep disturbance before exclusion of Zhang et al.’s studies [21, 23]. CI, confidence interval. [file CDD-26-318-s018.jpg]

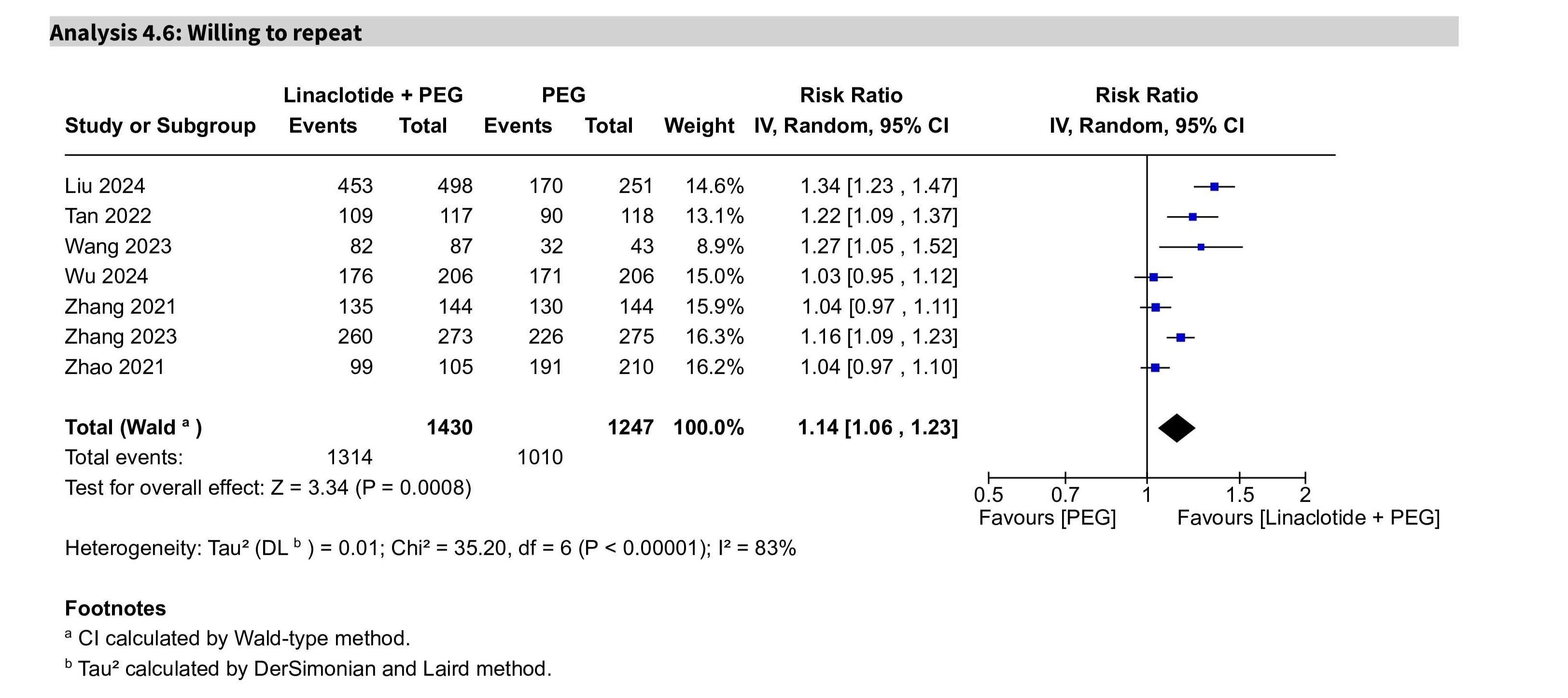

Supplement: Supplementary file 14 — Figure S13: Sensitivity analysis for willingness to repeat the colonoscopy procedure before exclusion of Liu et al.’ study [16]. CI, confidence interval. [file CDD-26-318-s009.jpg]

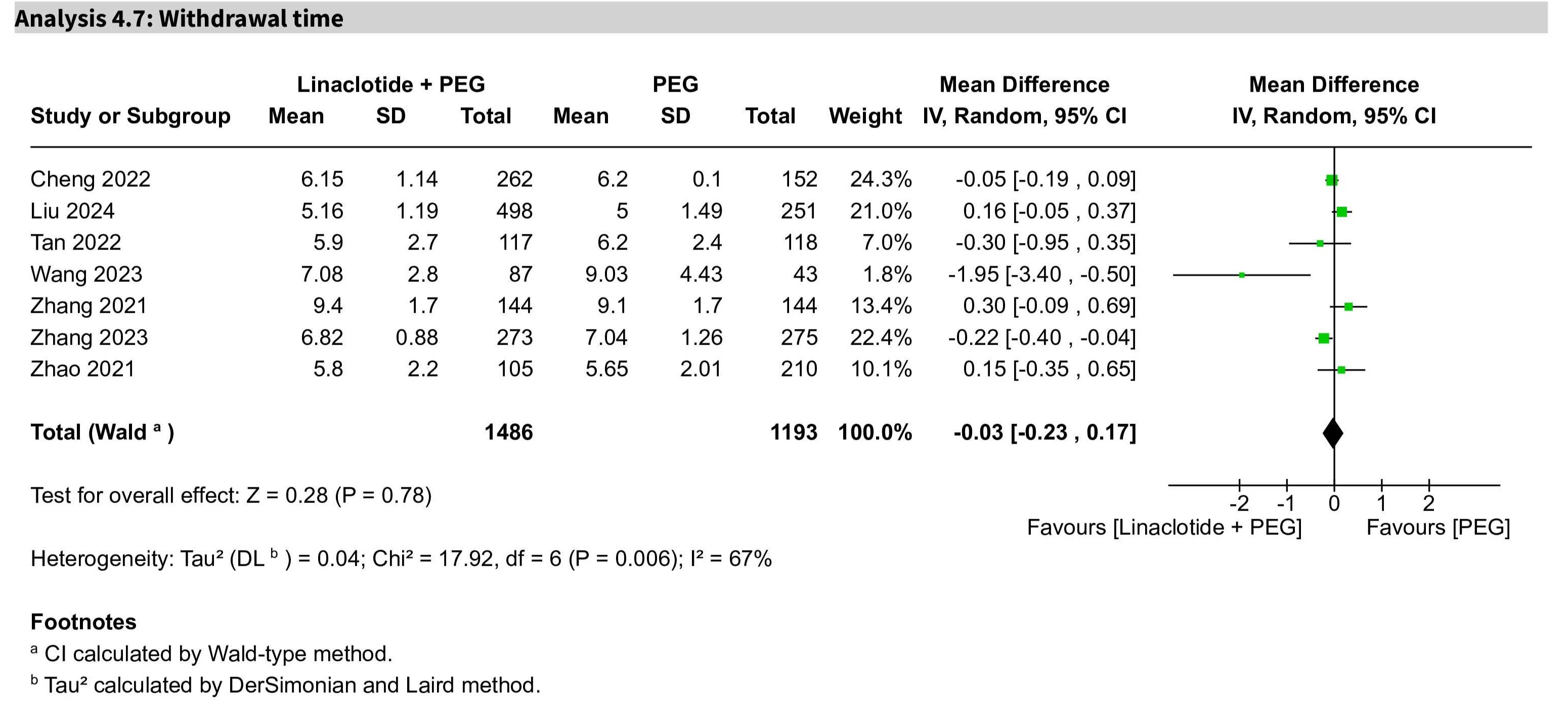

Supplement: Supplementary file 15 — Figure S14: Forest plot of withdrawal time comparing linaclotide plus polyethylene glycol [PEG] versus PEG alone for bowel preparation. CI, confidence interval. [file CDD-26-318-s008.jpg]

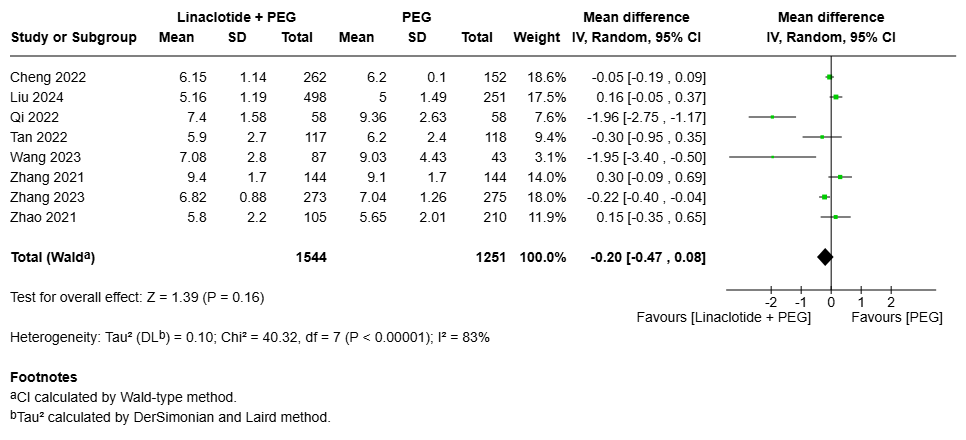

Supplement: Supplementary file 16 — Figure S15: Sensitivity analysis for withdrawal time comparing linaclotide plus polyethylene glycol [PEG] versus PEG alone for bowel preparation before exclusion of Qi et al.’s study [25]. CI, confidence interval. [file CDD-26-318-s013.png]

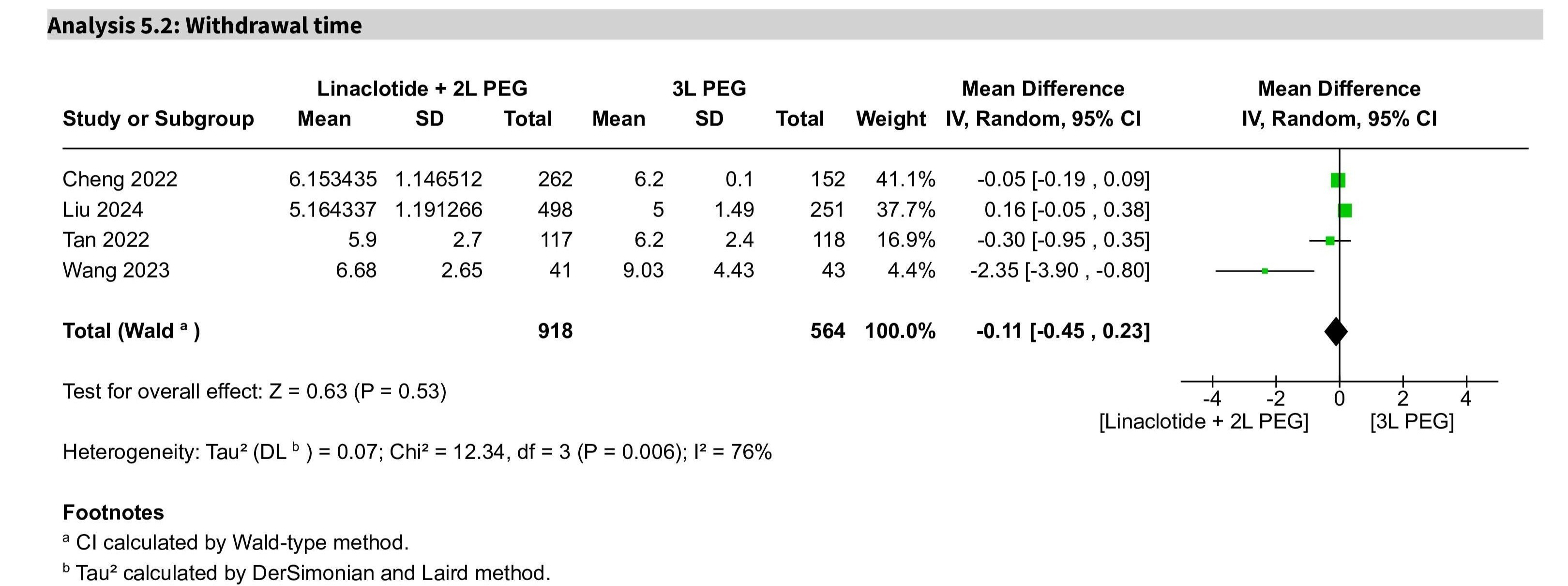

Supplement: Supplementary file 17 — Figure S16: Sensitivity analysis of withdrawal time (2‐L polyethylene glycol [PEG] plus linaclotide vs. 3‐L PEG) before exclusion of Wang et al.’s study [19]. CI, confidence interval. [file CDD-26-318-s014.jpg]

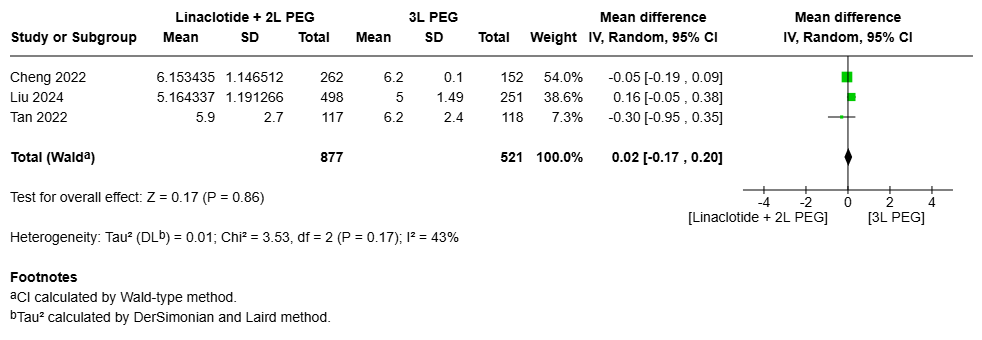

Supplement: Supplementary file 18 — Figure S17: Subgroup analysis of withdrawal time (2‐L polyethylene glycol [PEG] plus linaclotide vs. 3‐L PEG) after exclusion of Wang et al.’s study [19]. CI, confidence interval. [file CDD-26-318-s001.png]

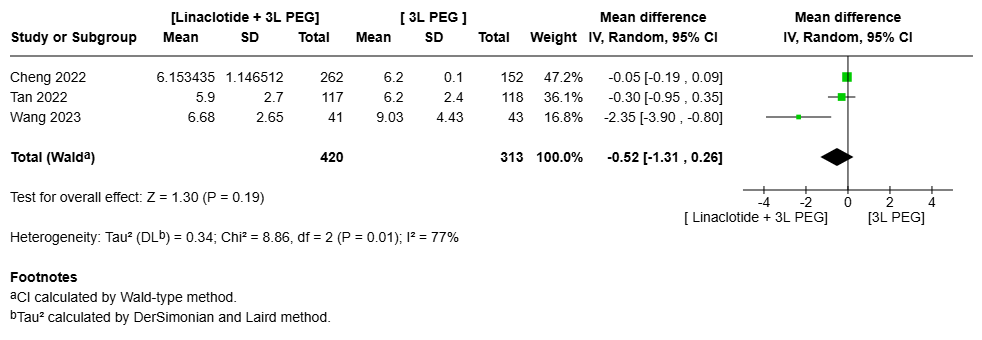

Supplement: Supplementary file 19 — Figure S18: Subgroup analysis of withdrawal time (3‐L polyethylene glycol [PEG] plus linaclotide vs. 3‐L PEG) after exclusion of Qi et al.’s study [25]. CI, confidence interval. [file CDD-26-318-s004.png]

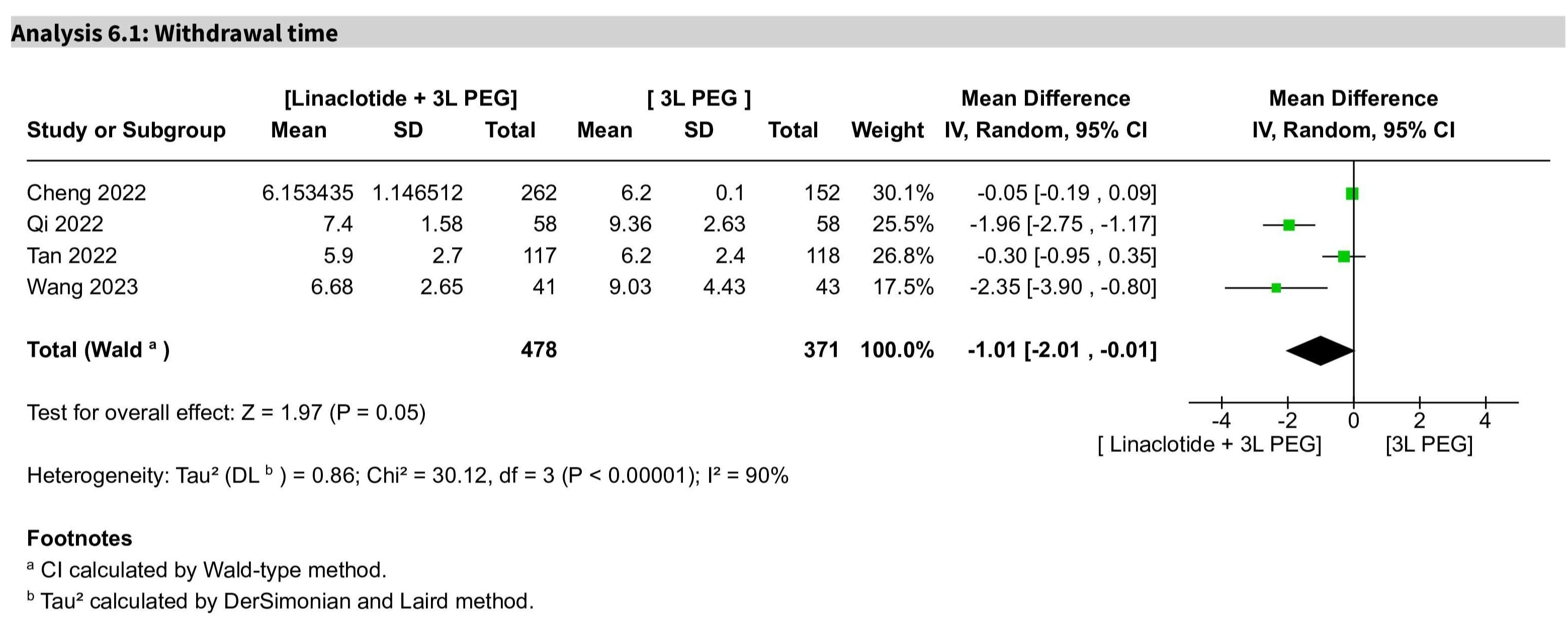

Supplement: Supplementary file 20 — Figure S19: Sensitivity analysis of withdrawal time (3‐L polyethylene glycol [PEG] plus linaclotide vs. 3‐L PEG) before exclusion of Qi et al.’s study [25]. CI, confidence interval. [file CDD-26-318-s011.jpg]
